# Annealing-Driven Phase Control Enables Plasmonic Tunability in Alloy Nanoparticles

**DOI:** 10.1021/acs.chemmater.5c01692

**Published:** 2025-10-21

**Authors:** Noah L. Mason, Anthony J. Branco, Sunhao Liu, Maëlisse Trancart, Sangmin Jeong, Connor S. Sullivan, Sarah S. Dawes, Smita Chatterjee, Sophia Manukian, Dugan Hayes, Lina Quan, Michael B. Ross

**Affiliations:** † 14710 University of Massachusetts Lowell, Department of Chemistry, Lowell, Massachusetts 01854, United States; ‡ 2331 University of North Carolina at Chapel Hill, Department of Chemistry, Chapel Hill, North Carolina 27599, United States; § 4260 University of Rhode Island, Department of Chemistry, Kingston, Rhode Island 02881, United States

## Abstract

A critical aspect of designing and realizing useful solid state materials is controlling phase and structure to tailor physical properties. While common for semiconductor and quantum materials, plasmonic materials have inhabited a narrow phase space typically comprising one or two elements, e.g., face-centered cubic metals. While this simplicity has enabled robust use and understanding of Au and Ag nanoparticles, it has also limited the design and manipulation of solid state properties. Here, we show that by tuning the phase and elemental composition of binary Au–Sn nanoparticles, the steady-state absorbance and ultrafast thermalization properties of plasmonic nanoparticles can be controlled. Solid state characterization suggests this is due to the dealloying of Sn and destabilization of the AuSn phase, leading to higher quality Au_5_Sn intermetallic phases alongside Au. Consequently, this work shows that phase control can profoundly influence the properties of plasmonic nanoparticles, providing important tunability for applications in catalysis, photothermal heating, and sensing.

## Graphical abstract

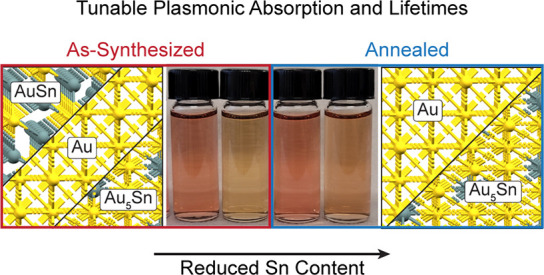

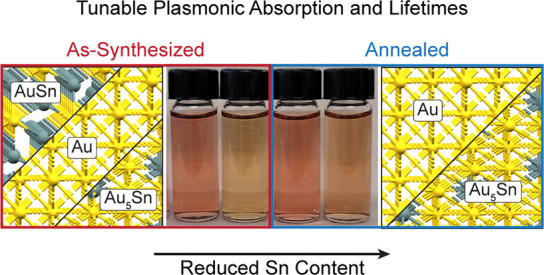

## Introduction

Controlling the phase and quality of solid state materials has enabled rapid advances in perovskite photovoltaics,^1^ two-dimensional supercapacitors,^2^ layered quantum materials,^3^ and magnetic mixed oxides.^4^ In contrast, the majority of plasmonics research to-date has focused on face-centered cubic metals.^5–7^ While recent work has shown that a richer set of nitrides and metals can exhibit plasmonic absorption, they are generally composed of a single phase without structural handles for property manipulation.^8,9^ These materials include nontraditional metals, such as Mg and Al,^10,11^ as well as nitrides and doped semiconductors.^9,12–14^ As such, there is a narrow scope within which one can control photophysical plasmonic properties using solid state structure and phase. While colloidal chemistry has enabled some synthesis of multimetallic alloys and intermetallics, their scope of properties remains relatively narrow.^15,16^ Realizing phase control is important for catalysis, where ordered phase(s) can show dramatically different activity due to finely controlled and ordered binding sites^17^ and for the manipulation of hot carriers and thermalization in metals where the phase-specific band structure can give rise to distinct electronic properties.^18,19^

Recently, we showed that alloying noble metal nanoparticles with post-transition metals can enable higher energy plasmonic absorption at visible wavelengths and into the ultraviolet.^20^ These synthesized nanoparticles comprise a variety of crystalline phases including face-centered-cubic (fcc) Au and Au_*x*_Sn_1–*x*_ substitutional alloys in addition to two intermetallic phases: trigonal Au_5_Sn and hexagonal AuSn. While only single phases are predicted thermodynamically by the bulk phase diagram, the rapid reduction of Sn leads to formation of a kinetic mixture of phases that vary in ratio as a function of Sn content.^20,21^ At the same time, the presence of these phases is associated with tunable higher energy localized surface plasmon resonances (LSPR), while higher intermetallic content led to damping and broadening.^22^

Here we show that engineering the phase composition and quality of Au–Sn nanoparticles can influence both their steady-state optical and time-resolved thermalization properties (Figure 1a). Specifically, mild annealing of Au/Au_5_Sn/AuSn nanoparticles reduces AuSn content in preference for Au_5_Sn intermetallic, as confirmed by X-ray diffraction (XRD) and ^119^Sn Mössbauer spectroscopy. The resulting annealed nanoparticles converge to a narrower LSPR lineshape, while time-resolved transient absorption (TA) reveals that the electron–phonon lifetimes remain within 25% of those of the Au nanoparticles while LSPRs can span a range of 40 nm.

**Figure 1:**
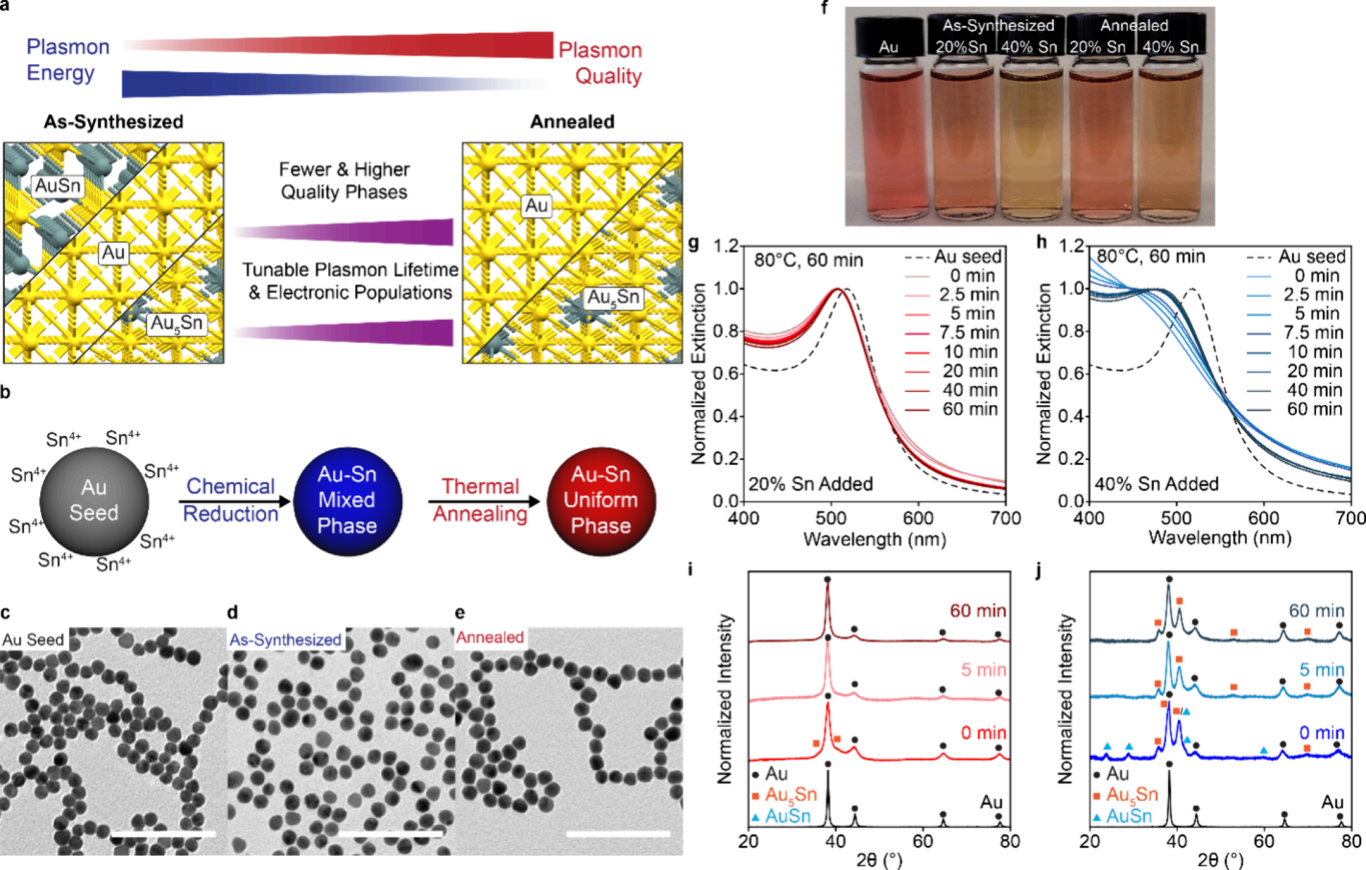
Phase dependent extinction in Au–Sn nanoparticles. (a) Tunability between plasmonic energy and quality by synthesizing nanoparticles with crystal structures of Au and Au–Sn intermetallics. (b) Scheme for seeded synthesis and thermal annealing of Au–Sn mixed phase nanoparticles to achieve a uniform phase. Transmission electron micrographs of (c) Au seeds, (d) Au–Sn mixed phase nanoparticles, and (e) Au–Sn phase uniform nanoparticles. Scale bars are 100 nm. (f) Photograph of Au seed, 20%, and 40% Sn-added nanoparticle solutions as-synthesized at 60 °C and after annealing for 1 h at 80 °C. Normalized extinction spectra of (g) 20% Sn and (h) 40% Sn-added Au–Sn nanoparticles taken at 0, 2.5, 5.0, 7.5, 10, 20, 30, and 60 min while annealing at 80 °C. XRD patterns of (i) 20% added Sn and (j) 40% added Sn at 0 min (as-synthesized), 5 min, and 60 min, compared to Au seeds. Diffraction pattern peaks are marked by fcc Au (black circle), trigonal Au_5_Sn (orange square), and hexagonal AuSn (blue triangles).

## Results and Discussion

To investigate the impact of phase mixing on the LSPR, a two-step process was carried out at ambient aqueous conditions (Figure 1b). First, ∼ 13 nm spherical nanoparticles were stirred with SnCl_4_ and polyvinylpyrrolidone and preheated to 60 °C in a water bath for 10 min. The solutions were removed and rapidly reduced using NaBH_4_ while stirring, followed by an additional 20 min at 60 °C to complete the reaction. Then, the colloidal solutions were allowed to cool to room temperature and were annealed in a water bath at 80 °C for up to 1 h (Methods). Transmission electron microscopy (TEM) shows that both the as-synthesized and annealed particles remain uniform and monodisperse (<10% coefficient of variance, COV) (Figure 1c-e, Table S1). For simplicity, a “high content” (40% Sn added) and “low content” (20% Sn added) Au–Sn colloid will be discussed in detail to showcase how annealing impacts nanoparticle characteristics for different Sn amounts.

Compared to the burgundy red Au seeds, the 20% Sn sample was orange-peach while 40% Sn was light tan. After annealing, these solutions changed color to peach and burnt-orange, respectively (Figure 1f). UV–visible spectroscopy more clearly highlights this change. First, both Sn-containing nanoparticles exhibit blue-shifted absorption compared to the Au seeds at 518 nm, with the low Sn content exhibiting a sharp LSPR at 502 nm and the high content having an LSPR at ∼ 452 nm. After annealing, the 20% added Sn exhibits a slight red-shift (509 nm) and narrowing (Figure 1g). Compared to the low-Sn sample, a more substantial change in LSPR lineshape is observed for the 40% Sn-added nanoparticles, with a transition from a broad absorption feature to a less damped LSPR (484 nm) after 1 h of annealing (Figure 1h). X-ray diffraction (XRD) of the 20% added Sn reveals diffraction peaks that correspond primarily to fcc Au and the formation of Au_*x*_Sn_1–*x*_ consistent with our prior work that suggest that substitutional alloying leads to blue-shifting at lower Sn contents.^20^ A subtle reflection at 40.1° is also observed corresponding to Au_5_Sn, however this peak is no longer observed after 5 min of annealing (Figure 1i). In the 40% added Sn case, fcc Au/Au_*x*_Sn_1–*x*_, Au_5_Sn, and AuSn intermetallic are observed.^20^ The diffraction peaks that correlate with AuSn disappear after only 5 min of annealing (Figure 1j); this correlates with the most dramatic change in LSPR lineshape. In addition, the emergence of higher order reflections (52.5° and 69.6°) and sharper primary peaks suggests higher quality Au_5_Sn — with larger and more uniform crystallites — compared to the as-synthesized phases.^23^

Other added Sn amounts (10% and 30%) were investigated which exhibited analogous trends upon annealing. For 10% Sn added, a slight blue-shift is observed (514 nm) with a sharp LSPR, while 30% Sn added has a broader LSPR at 475 nm. Annealing drove the LSPR of 10% Sn to one that is nearly superimposable to the starting Au nanoparticles while 30% Sn red-shifts to 500 nm (Figure S1). XRD patterns for 10% and 30% Sn show fcc Au/Au_*x*_Sn_1–*x*_ reflections and Au/Au_5_Sn reflections, respectively, without substantive visual change during annealing (Figure S2).

Quantifiable changes in the Au/Au_5_Sn/AuSn phase content were observed for all samples with >10% Sn added as measured by Rietveld refinement (Figures S3–5). For the 20% Sn-added sample, the Au_5_Sn content decreased upon annealing from 12 to 0% and for the 30% Sn-added sample it decreased from 28 to 20%. In contrast, the Au_5_Sn content increased in the 40% Sn-added nanoparticles upon annealing from 34% to ∼ 60%, while the AuSn content decreased from 39% to 0% (Figures S3–5). Similar effects are observed at other annealing temperatures (Figures S6–13). Annealing at 95 °C removes AuSn within 2 min while room temperature annealing takes ∼ 12 h to remove AuSn (Figures S14, 15). In all cases, the decrease of AuSn content is accompanied by an increase in Au_5_Sn content, after which the Au_5_Sn content slowly decreases over the duration of the remaining annealing. This suggests that annealing can convert between AuSn and Au_5_Sn phases, after which dealloying of Sn from the nanoparticle occurs.^21,24,25^

The conversion of intermetallic and dealloying was further corroborated by altering the reaction temperature to influence the phase formation and stabilization. We hypothesized that altering the synthesis temperature would alter the kinetic stabilization of alloy phases. The synthesis of Au–Sn nanoparticles was performed at 40 and 80 °C with 40% Sn-added, analogous to Figure 1. It was observed that AuSn content and blue-shifting both increased at lower synthesis temperatures (Figure 2a, c, e). After annealing for 1 h at 80 °C, the UV–visible spectra and XRD patterns converged to be nearly superimposable with the 80 °C synthesis (Figures 2b, d, f, S16). Rietveld analysis quantitatively confirms these trends with similar changes and more pronounced interconversion of AuSn-Au_5_Sn–Au as were observed in the 60 °C case described above (Figures S17–19). Higher amounts of Sn added seem to yield less thermodynamically stable phase mixtures of AuSn/Au_5_Sn due to the kinetically driven, diffusion-based synthesis of these nanoparticles.^26–30^ This is consistent with what would be predicted by the bulk phase diagram, i.e. stabilization of fewer colocalized phases. Overall, temperature plays a profound role in controlling the intermetallic phases for a given Sn amount added during synthesis.

**Figure 2:**
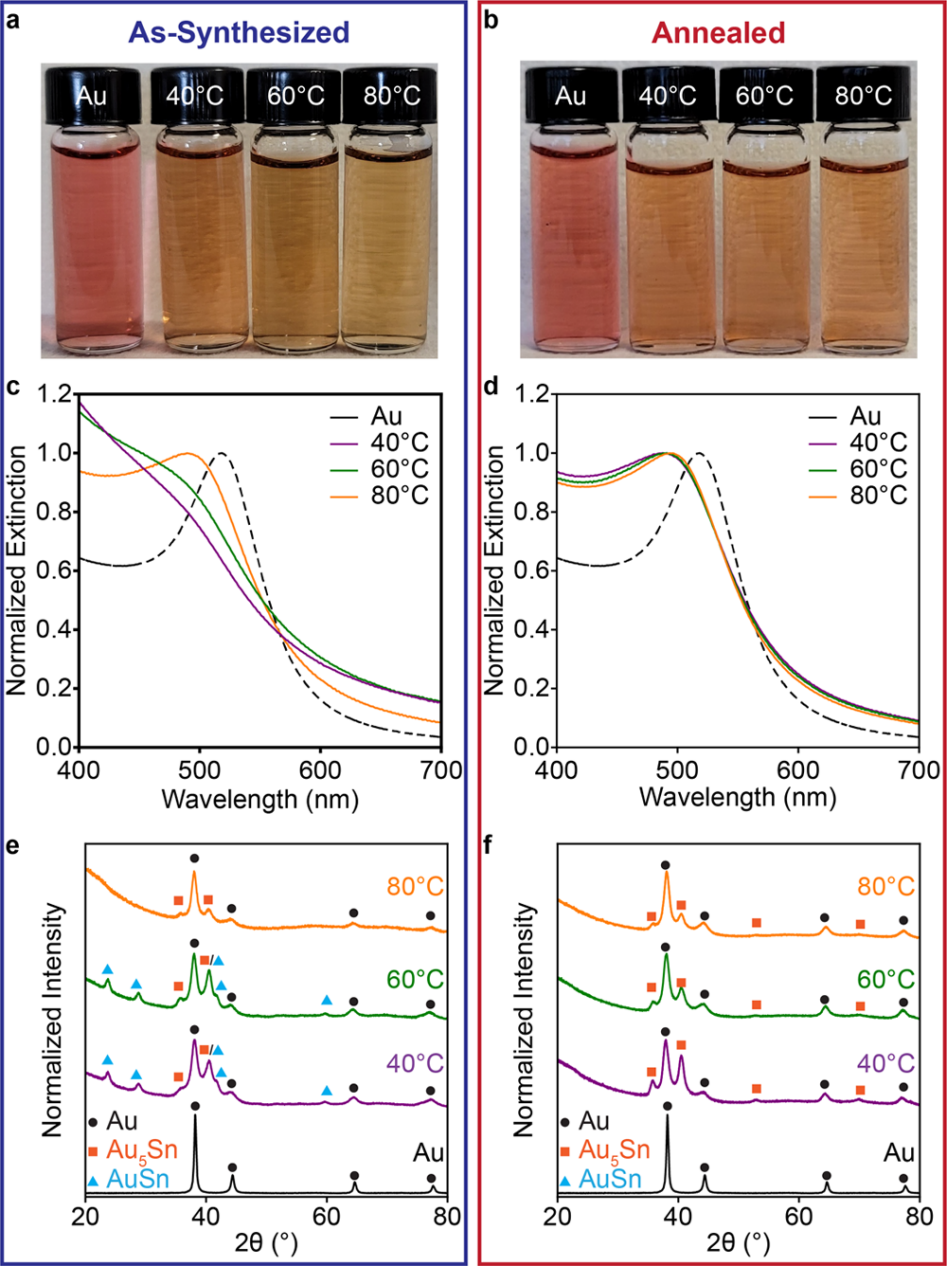
Temperature controlled phase stabilization, mixing, and convergence in physical properties. Photographs of (a) As synthesized 40% Sn-added Au–Sn nanoparticle colloids synthesized at 40, 60, and 80 °C and (b) annealed 40% Sn-added Au–Sn nanoparticle colloids achieved after annealing at 40, 60, and 80 °C for 1 h. Normalized extinction spectra of Au seeds and Au–Sn nanoparticle solutions (c) before and (d) after annealing at 80 °C for 1 h. X-ray diffraction patterns of Au seeds and Au–Sn nanoparticle solutions (e) as-synthesized and (f) after annealing at 80 °C for 1 h. Diffraction pattern peaks are marked by fcc Au (black circle), trigonal Au_5_Sn (orange square), and hexagonal AuSn (blue triangles).

To better understand the impact of annealing on the structure and composition of Au–Sn nanoparticles, more comprehensive characterization was performed on the nanoparticles synthesized and annealed at 60 °C (Figure 1). High-resolution transmission electron microscopy (HRTEM) shows that as-synthesized and annealed nanoparticles remain uniformly crystalline with little change to overall morphology (Figure 3a,c,e,g). In addition, a 1–2 nm SnO_2_ shell can be seen around each nanoparticle after ambient exposure. Fast Fourier transform (FFT) image analysis of the individual crystallites reveals similar changes in crystalline phases as is observed by XRD (Figure 3c,d,f,h, Figure S20). The 20% Sn added as-synthesized samples are seen to contain Au (111) and Au_5_Sn (110) planes while after annealing only Au-specific (111) and (200) planes are observed. In the 40% Sn added samples, Au (111), Au_5_Sn (110), and AuSn (100) planes are all observed, whereas after annealing only Au and Au_5_Sn are seen. These data confirm that the crystalline phases coexist within a single nanoparticle in few-nm domains. Dark-field scanning transmission electron microscopy (DF-STEM) coupled with energy-dispersive X-ray spectroscopy (EDS) further shows that Sn content is consistent through the volume of each particle both before and after annealing (Figures S21, S22). Electron micrographs of the nanoparticles synthesized at 40 and 80 °C (Figure 2) also show similar structural features and detectable Sn content in all cases (Figures S23–S25).

**Figure 3:**
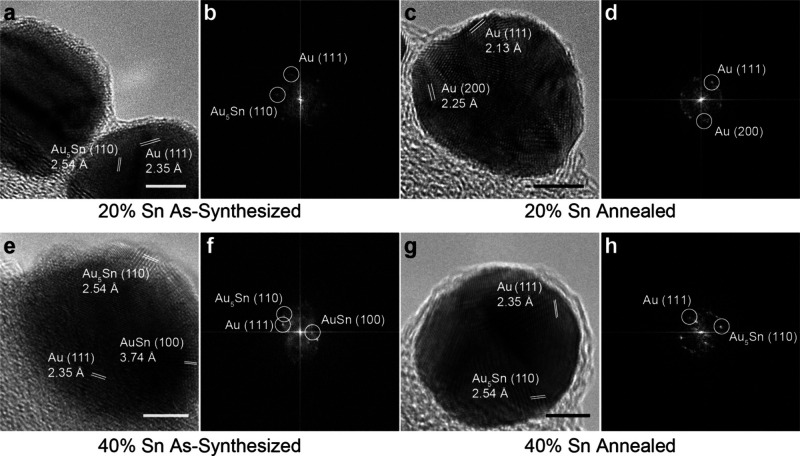
Au–Sn electron microscopy characterization. High-resolution transmission electron microscopy images and fast Fourier transform analysis for a 20% Sn-added Au–Sn nanoparticle (a, b) as-synthesized and (c, d) after annealing and a 40% Sn-added Au–Sn nanoparticle (e, f) before and (g, h) after annealing. All scale bars are 5 nm.

Although similar nanoscale morphologies are maintained after annealing, compositional characterization by EDS and X-ray photoelectron spectroscopy (XPS) shows that annealing reduces the Sn content. XPS shows that annealing reduces the Sn content significantly (Figures S26, S27, Table S2). For nanoparticles synthesized at 60 °C, surface Sn content decreases from 38.24% (20% Sn added) and 54.37% (40% Sn added) to 1.04 and 3.30% after annealing. STEM-EDS reveals that ∼ 10–15% Sn remains in the nanoparticle volume regardless of starting Sn content (Table S2). Inductively coupled plasma optical emission spectroscopy (ICP-OES) of the as-synthesized nanoparticles, the supernatant after annealing, and the annealed nanoparticles reveal that the annealing process itself drives Sn out of the nanoparticles and into the supernatant, reducing the Sn content (Figure S28). A putative mechanism for this would be Sn oxidation that solubilizes Sn cations. This mechanism is consistent with the observed phase changes also occurring at room temperature, albeit at a slower rate than when annealed. This plausible mechanism could be driven either by Au diffusion along grain boundaries or Sn diffusion through either the lattice or along grain boundaries, both of which have been observed previously.^27–29^ The higher quality XRD data after annealing suggest that any defect formation is transient, given that higher quality crystallites are observed in the final nanoparticles. Analogous trends are observed for the synthesis at 40 °C, while synthesis at 80 °C has little surface Sn content observed in XPS both as-synthesized and annealed (Figures S29–S30). These data reveal that the Sn content in the nanoparticles is reduced upon annealing, particularly at the surface.

Alongside high-resolution TEM which revealed uniform Sn coverage and a thin SnO_2_ shell, ^119^Sn Mössbauer spectroscopy was used to directly investigate the Sn-containing phases. Using SnO_2_ as a reference, spectra were collected for 20% and 40% Sn samples before and after annealing (Figures S31–S34, Materials and Methods). For the 20% Sn sample, two peaks were observed at ∼ 0 mm/s and ∼ 2 mm/s (Figure 4a, Figure S35). After annealing, the peak at ∼ 2 mm/s remains largely unchanged while the 0 mm/s feature decreases in amplitude (Figure 4b), indicating a reduced amount of oxide. A similar phenomenon is observed for the 40% Sn-added samples, while an additional feature is observed at ∼ 0.6 mm/s that disappears after annealing (Figure 4c, d). No additional features were observed at higher velocities and the spectra are consistent across samples from separate preparations (Figures S33–34, 36). Previous reports show that Au_*x*_Sn_1–*x*_ solid solution appears as a singlet at ∼ 2 mm/s.^31^ For oxide species, SnO_2_ is expected at 0 mm/s (as the spectra are referenced to bulk SnO_2_), while the absence of a distinct quadrupole-split doublet at ∼ 2.5 mm/s rules out the existence of SnO. The remaining phases AuSn and Au_5_Sn were assigned to the ∼ 0.6 mm/s peak and ∼ 2 mm/s peak respectively based on XRD analysis, and the temperature-dependent phase behavior described above. All Mössbauer fit parameters are collected in Table S3.

**Figure 4:**
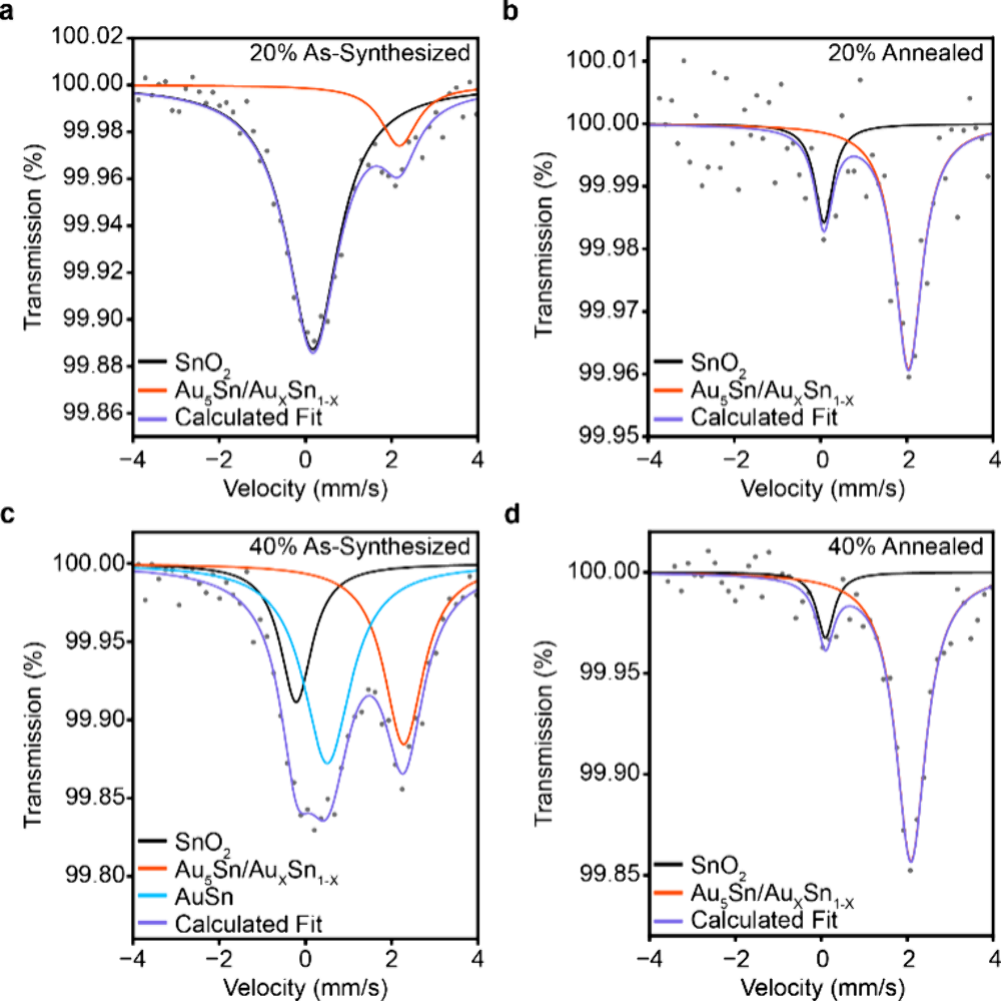
Sn-phase analysis in Au–Sn nanoparticles by ^119^Sn Mössbauer spectroscopy. Binned Mössbauer spectra of 20% Sn-added Au–Sn nanoparticles (a) as-synthesized and (b) after annealing and 40% Sn-added Au–Sn nanoparticles (c) as-synthesized and (d) after annealing.

During refinement of the synthesis, it was observed that some variability in the nanoparticle composition is observed, particularly for added Sn amounts >30%. This was hypothesized to be due to the interplay between reaction temperature, reduction, and diffusion, each of which could impact phase formation at a given Sn amount. Annealing, however, could eliminate this phase variability by allowing a convergence to the most favorable phase content. To investigate this, repeatability experiments were performed in replicate by 10 different individuals as a part of an on-sight launch program for incoming undergraduates, *CatalyzeUML.* 20% and 40% added Sn nanoparticles were synthesized at 60 °C and their optical properties were characterized by UV–visible spectroscopy. They were then annealed at 80 °C for 1 h and analyzed after by TEM, XRD, and UV–visible spectroscopy (Figure 5).

**Figure 5:**
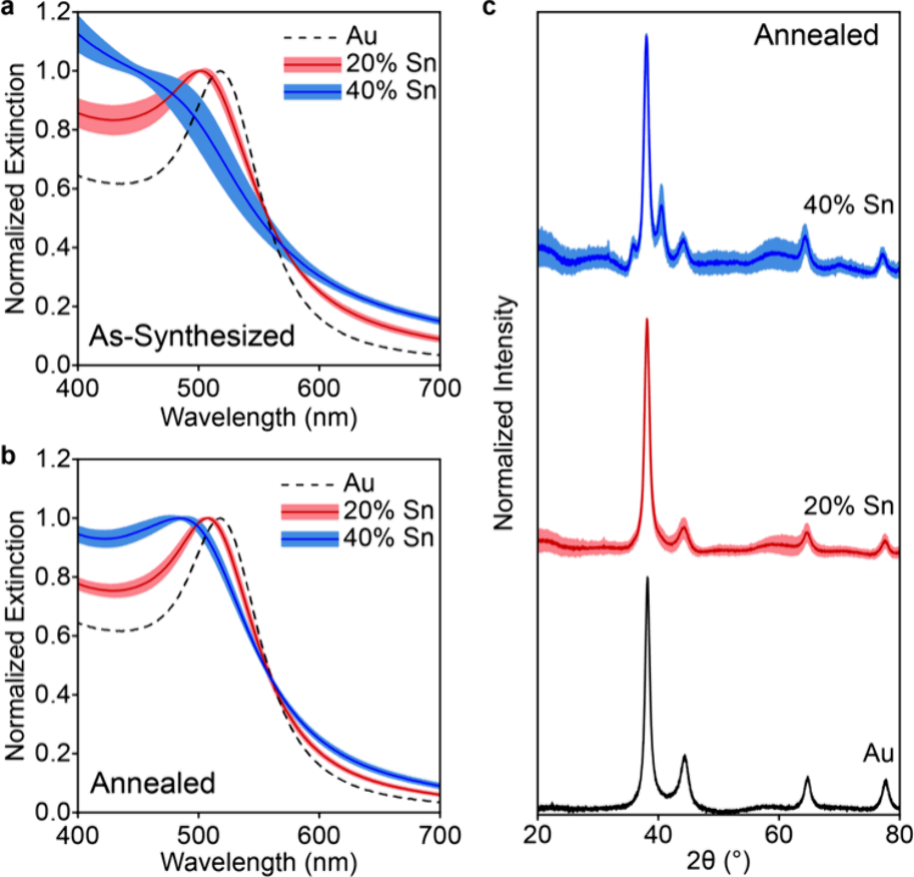
Uniform phase repeatability upon thermal annealing. Normalized extinction of Au seeds and the resulting average (line) and standard deviation (shaded region) of 10 replicate 20% and 40% added Sn spectra (a) as-synthesized and (b) after annealing at 80 °C for 1 h. (c) XRD pattern for Au seeds and average (line) and standard deviation (shaded region) of 10 replicate 20% and 40% added Sn patterns after annealing at 80 °C for 1 h.

The extinction spectra were measured for each sample and fit by Lorentzian curves to quantify the LSPR location and the linewidth, the latter of which relates to the plasmonic quality as well as the timescales of plasmonic excitation.^32,33^ The as-synthesized extinction spectra show some variation between replicates: ± 4.2 and ± 13.7 nm in LSPR maxima and ± 35.6 and ± 79.4 meV in line width for 20 and 40% Sn, respectively, though the deviation in as-synthesized 40% Sn is limited to the only three samples which could be fit. (Figures 5a, S37). The annealed solutions showed a smaller standard deviation for 20% Sn added and a quantifiable deviation for all 40% annealed samples: ± 2.3 and ± 8.0 nm in LSPR maxima and ± 15.3 and ± 82.7 meV in linewidth (Figures 5b, S38). Minimal sample-to-sample phase differences are observed after annealing; the 40% Sn added show a mixture of Au_5_Sn and Au_X_Sn_1‑X_ phases while the 20% Sn added only shows fcc phases (Figures 5c, S39). TEM of annealed nanoparticles shows that the size and shape of the nanoparticles remain uniform across the replicates (Figures S40–41, Table S1). Overall, this shows that annealing converges the colloidal dispersion to a more uniform phase distribution that is reflected in the LSPR across replicates, mitigating variability in the synthesis.

At higher Sn content, a wide distribution in linewidths is observed and seven of the replicates were unquantifiable due to significant damping of the LSPR (Figures 6a, S42). At lower Sn content, the linewidths show less spread and were readily quantified. In all cases, annealing red-shifted and narrowed the LSPR. For comparison, the intrinsic nonradiative loss native to bulk Au and Sn was calculated from the dielectric functions (Materials and Methods).^34–36^ The linewidth of the Au seeds is superimposable onto the intrinsic nonradiative loss curve, which is reasonable for small spherical Au nanoparticles of this radius.^37,38^ This suggests that the dephasing of the LSPR is well-described by bulk processes. In contrast, the Au–Sn alloy nanoparticles all have LSPRs below the nonradiative loss limit, i.e. with linewidths that are more intrinsically narrow than would be possible for a pure Au nanoparticle. While an approximation — because Au cannot intrinsically achieve LSPRs at wavelengths shorter than ∼ 520 nm — this does suggest that the intrinsic electronic and physical properties are distinct and modified due to the integration of Sn-enriched phases. Sn, notably, has less intrinsic loss at these wavelengths.^8,32,36^

**Figure 6:**
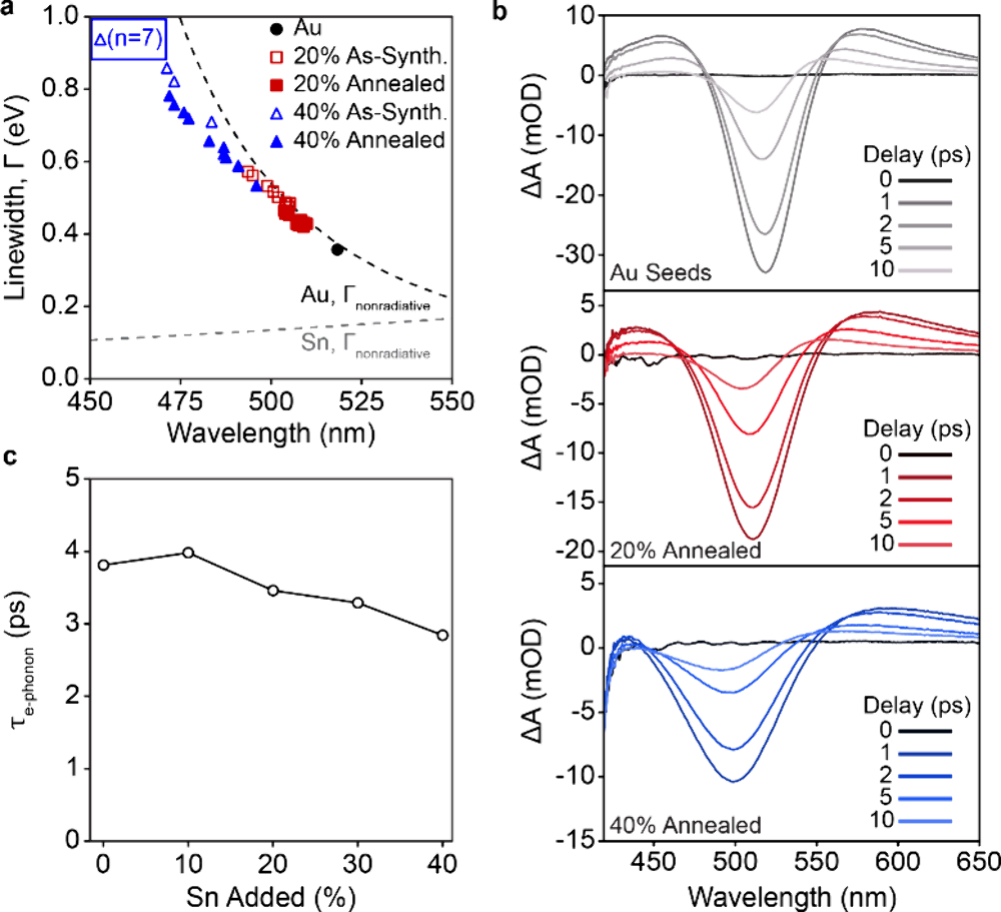
Linewidth and LSPR lifetime analysis of phase pure Au–Sn nanoparticles. (a) Linewidths for each 20% and 40% Sn-added replicate as-synthesized and after annealing at 80 °C for 1 h. The theoretical nonradiative broadening of metallic Au and Sn (dashed curves) are included for comparison. (b) Transient absorption spectra of Au seeds and annealed 20 and 40% Sn-added nanoparticles with 400 nm excitation and probing faster than 10 ps. (c) Calculated electron–phonon lifetimes as a function of Sn amount added for the annealed samples and a gold seed control.

The plasmon dephasing and quality were then directly quantified as a function of annealing and Au–Sn composition. The LSPR in metals typically dephases at time scales on the order of tens of femtoseconds through radiation or scattering with defects and the nanoparticle surface.^18,39,40^ In nanoparticles of this size, radiation damping is minimal and the primary decay mechanisms are nonradiative.^39^ The dephasing time can be directly related to the plasmonic linewidth by *T* = 2*ℏ*/Γ. It is observed that all Sn-containing nanoparticles have dephasing times shorter than the pure Au seeds (Table 1).^34,38^ This could be explained by increased loss in Au at shorter wavelengths, loss mechanisms introduced by Sn addition, or by the formation of intermetallics.^41^ Notably, annealing—which removes AuSn phase and increases the crystalline quality (Figure 5)— extends the dephasing times by ∼ 15–20% (Table 1). It is also worth noting that there is a path dependent aspect to structural and optical properties that further supports the impact of phase on the LSPR. The 20 and 40% Sn-added nanoparticles start with dramatically different Sn contents, after annealing they are within ∼ 6% (Table S2), and yet their LSPRs still differ. The primary structural difference that remains is that the 20% annealed contains only fcc phase while the 40% annealed contains Au_5_Sn and fcc. These are consistent with our prior theoretical modeling work that suggests either substitutional alloying or a core–shell intermetallic-Au motif could lead to blue-shifting of the LSPR.^20^ Resonance quality factors, *Q*, were also calculated by *Q*=Γ/*E*_LSPE_.^38^ Higher Q-factors relate to enhanced near fields, long lifetimes, and less plasmon damping. Similar 15% enhancements were observed in both annealed materials compared to their as-synthesized states.

**Table 1:** Impact of Annealing on Plasmon Quality and Lifetime

Sample	LSPR Location (nm)	Linewidth, Γ (meV)	Dephasing Time, *T* (fs)	Quality Factor, *Q*
Au Seed	518	332	3.97	7.2
20% Au–Sn As-Synth.	501	499	2.64	5.0
40% Au–Sn As-Synth	458	793	1.66	3.4
20% Au–Sn Annealed	507	425	3.10	5.8
40% Au–Sn Annealed	483	658	2.00	3.9

While the linewidth relates to the LSPR quality and the dephasing of coherent plasmonic oscillation at femtosecond time scales, it does not provide insight into the thermalization of the hot carrier distribution resulting from the excitation. To more directly understand the dependence of plasmon thermalization on structure, picosecond transient absorption (TA) spectroscopy was carried out to characterize the temporal photophysical properties of the annealed nanoparticles as a function of added Sn content (Figure 6b-c).^39^ Specifically, ultrafast TA provides specific insight into the cooling of the hot electronic distribution through electron–phonon scattering with the lattice.^18,39,42^ In all TA spectra, a large ground state bleach (GSB) signal was observed near the LSPR maximum at around 500 nm after which the decay curve was fit (Figures 6b, S43). For all nanoparticle compositions, the electron–phonon time constants ranged from ∼ 3–4 ps (Figure 6c, Table S4). For the annealed samples, the time constant for the lower Sn content nanoparticles were within 5–10% of the pure seeds, and that of the highest 40% Sn-added sample is within 25%. These data, combined with the narrowed steady-state linewidths, suggest that phase engineering can be used to manipulate the photophysical properties of the nanoparticles such that high-quality LSPRs and desirable thermalization profiles can be achieved over a wide spectral range.

## Conclusions

In mixed phase plasmonic nanoparticles, mild annealing is shown to create a more uniform, high-quality phase content that in turn narrows the plasmon linewidth, enables independent tuning of the thermalization properties, and converges variability in the synthesis. Rapid chemical reduction is hypothesized to stabilize kinetic intermetallic phases that mild aqueous annealing drives out of the system to a desirable Sn content with varying final crystal structures and oxide content. The direct relationships formed between phase content, plasmon quality, and thermalization lifetimes highlight how direct manipulation of phase provides a new means for controlling plasmonic properties not possible in unary metals. These results raise important opportunities for understanding how composition, atomic structure, and nanoscale phase mixing can together influence plasmonic properties. Higher resolution structural characterization coupled with photophysical measurements with few-fs temporal resolution could reveal the critical early dynamics that lead to high-quality LSPRs in these systems. Meanwhile, aberration-corrected electron microscopy could further allow clear identification of all phase boundaries and defects which could further clarify the key phase and structural motifs that drive plasmonic tunability and damping. If coupled with selected-area electron diffraction (SAED), this could provide better phase information across the ensemble of the material. This would be best supported by enhanced synthetic procedures that can create both phase-pure intermetallic nanoparticles in addition to accessing the two Sn-rich intermetallic phases, AuSn_2_ and AuSn_4_, that are predicted in the bulk. Theoretical work will aid in understanding the mechanisms by which phase mixing can influence plasmon dynamics and quality in new ways.

This work has important implications for designing novel plasmonic materials for next-generation applications in catalysis, heating, distillation, soldering, and optical electronics.^17–19^ The ability to use alloying and phase design to control—and even improve— photophysical properties provides a new strategy for plasmonic materials, reminiscent of strategies common for other solid state material devices. The specific use of post-transition metals here allows one to create higher energy and narrower LSPRs compared with the noble metals. Many of the post-transition metals, and Sn specifically, are active toward electrochemical CO_2_ reduction.^43^ Overall, this approach allows one to manipulate structure in multimetallic mixtures, expanding the library of high-energy absorbing plasmonic nanomaterials with novel phase compositions.

## Materials and Methods

### Materials

Hydrogen tetrachloroaurate (III) trihydrate (HAuCl_4_·3H_2_O, 99.99%, Alfa Aesar), trisodium citrate dihydrate (99%, Alfa Aesar), tin­(IV) chloride (SnCl_4_·, 99.99%, Alfa Aesar), poly­(vinylpyrrolidone) (PVP, MW = 40 000, Alfa Aesar), and sodium borohydride (97+%, Alfa Aesar) were used without further purification and all solutions were prepared with 18.2 MΩ resistivity water.

### Synthesis of Au Seeds

Gold nanoparticle seeds were prepared using an adapted Turkevich synthesis.^44,45^ In a 100 mL round-bottom flask set in a heating mantle held at 130 °C with rapid stirring (650 rpm). 58.56 mL of water and 1.2 mL of 10 mM HAuCl_4_ were brought to reflux. Once at reflux, 480 μL of 100 mM sodium citrate solution was rapidly injected. The solution was heated for 8 min after citrate addition, after which a color change to purple followed by reddish-pink was observed. The solution was then removed and allowed to cool to room temperature before use. The Au seeds were used as synthesized at ∼ 0.7 O.D.

### Synthesis of Au–Sn Nanoparticles

Synthesis of Au–Sn nanoparticles followed a seed-mediated approach.^20^ 5 mM SnCl_4_·solution (according to the desired molar percent composition) was added to 3 mL of Au seeds in 20 mL glass scintillation vials with vigorous stirring at room temperature. Then, 10 wt % PVP solution was added according to a fixed ratio of 0.06:1 PVP to total molar metal content (Au and Sn), after which water was added to bring the total reaction volume to 4 mL. After this, the solutions are placed in a 40 °C, 60 °C, or 80 °C water bath for 10 min. The solutions were removed after 10 min and placed under vigorous stirring for the addition of freshly prepared 260 mM NaBH_4_ solution according to a fixed 30:1 ratio of reducing agent to total molar metal content (Au and Sn) in solution. The vials were then placed back into the same water bath for 20 min, after which they were removed and allowed to cool back to room temperature for ∼ 15 min before characterization.

### Thermal Annealing of Au–Sn Nanoparticles

Thermal annealing was carried out by placing as synthesized colloidal samples into a preheated water bath at the desired annealing temperature. After the desired annealing time, samples were removed from the water bath and quenched in an ice bath until they returned to room temperature for further characterization.

### UV–Visible Spectroscopy

UV–visible spectra were recorded with an Agilent Cary 100 spectrophotometer. Sample measurements were taken on room temperature samples using a quartz 1 cm path length cuvette without the need for further sample preparation.

### Transmission Electron Microscopy

Transmission electron microscopy was performed using a Phillips CM-12 and HR-TEM was performed using a JEOL, JEM-2100Plus electron microscope. All imaging was performed at 200 kV. Samples were prepared for imaging by centrifugation at 5600 r.c.f. for 10 min, followed by removal of supernatant and resuspension in water. A second centrifugation at 8600 r.c.f. for 8 min was run and the supernatant was removed and discarded. Approximately 20 μL of water was added to the pellet to disperse the pellet and ∼ 5 μL of concentrated product was then drop-cast onto Cu Carbon Type-B grids and Cu Lacey Carbon 200 mesh grids (for HR-TEM/STEM imaging) from Ted Pella.

### Powder X-ray Diffraction and Phase Analysis

Powder X-ray diffraction (XRD) measurements were performed on a Rigaku Miniflex X-ray diffractometer using Cu Kα (λ = 1.5418 Å) radiation in the 2θ range of 10 – 90°, and a scan rate of 1° min^–1^. 8.0 mL of colloidal sample was concentrated to ∼ 200 μL and drop-cast onto a zero background Si sample holder (Rigaku) and dried at room temperature. Individual crystal phases were indexed using the crystallographic open-source database. Specific material reference numbers include: Au (9013036), Au_5_Sn (1510571), and AuSn (1510301). Rietveld refinement was performed using the Rigaku software fitting to minimize the residual. One challenge specific to these systems is that Au_*x*_Sn_1–*x*_ cannot be used as a fitting parameter, only bulk Au, and as such there is more pronounced residual at the (111) fcc reflection in all cases. However, indexing alone is sufficient to support the significant phase changes observed in these systems.

### STEM-EDX Compositional Analysis

Energy dispersive X-ray spectroscopy-coupled scanning transmission electron microscopy (STEM-EDX) was performed using a JEOL JEM-2100Plus scanning transmission electron microscope (STEM) equipped with a corresponding JEOL normal dark-field detector, and a JEOL Dry SD100GV Silicon Drift EDX detector. Wide-field and Dark-field STEM imaging was performed at magnifications between 800,000 and 2,000,000. EDX analysis was carried out using the Analysis Station software package to assess the composition of Au and Sn using Sn L-edge and Au M-edge to quantify the atomic ratios. Three spots on the same prepared sample were analyzed using dwell times between 0.01 and 0.03 ms and were collected for between 20 and 25 min with probe tracking on. These composition ratios were determined directly with the aid of the Analysis Station software package without further processing.

### Lattice Plane Identification with Fast Fourier Transform (FFT)

Fast Fourier Transform (FFT) with lattice spacings was manually measured using the HR-TEM integrated software DigitalMicrograph (Gatan, Inc.) from HR-TEM images of Au–Sn samples at a scale of 5 nm. First, a rectangular frame was input on the TEM image to measure the lattice spacing, and FFT data was generated. The FFT pattern was then analyzed to identify the characteristic diffraction peaks (Bragg scatter/diffraction) corresponding to the crystal planes of the respective phases. By selecting the appropriate FFT pattern, respective intensity distributions were subsequently generated. The measured lattice constant was determined by averaging diffraction peaks by the number of lattice spacings. These lattice distances were compared with theoretical lattice spacings from XRD data of Au, Au_5_Sn, and AuSn. The comparison provided confirmation of the material’s crystal structure.

### Quantitative Analysis by Inductively Coupled Plasma Optical Emission Spectroscopy (ICP-OES)

Sample preparation was performed as described previously and performed on an Agilent 5110 ICP-OES system with Agilent’s ICP Expert software package. For each sample, analysis was performed on the as-prepared colloid (without any washing), the final “incorporated” nanoparticle colloid (after centrifugation and rinsing off excess reactants), and two supernatants which correspond to each centrifugation steps. This allowed assessment of how much Sn was incorporated throughout the standard synthesis processes and also annealing processes. All colloid or supernatant aliquots were allowed to fully dry in a vacuum oven (under only vacuum) and were digested with 200 μL of aqua regia (using trace metal grade HCl and HNO_3_) and 800 μL of ultrapure water in the sample container as were dried. Once fully digested and brought to 1000 μL total volume, intrasample subsets were diluted to 5 mL, and triplicates of ICP-OES were measured. Samples and blanks were compared to calibration curves using a multielement ICP standards containing both 10 ppm stock Au and 10 ppm stock Sn. Relative at.% of Sn in each sample were calculated after retrieval of concentrations from ICP-OES with respect to Au.

### X-ray Photoelectron Spectroscopy

Samples for X-ray photoelectron spectroscopy (XPS) were prepared by concentrating 2.0 mL of colloidal sample to ∼ 50 μL and subsequently drop-casting 2 μL of concentrated product onto an Si wafer. This was allowed to dry at room temperature, after which another 2 μL was added on top of the previous sample. Layering is continued in this fashion until a homogeneous metallic sheen can be seen (∼20 repetitions). XPS was performed using a PHI Versaprobe II using an Al Kα X-ray radiation, and XPS peaks were calibrated to the C 1s peak at 284.8 eV.

### ^119^Sn Mössbauer Spectroscopy

Samples for ^119^Sn Mössbauer Spectroscopy were prepared by concentrating 800 mL of colloidal sample to ∼ 200 μL and drop casting onto Kapton tape to give a circular film ∼ 4 mm in diameter. Each concentrated sample was then mounted to a lead sample holder with a 4 mm aperture and measured at room temperature using an M6 Resonant γ-ray spectrometer (SEE Co.) with a 1024-channel Kr/CO_2_ proportional counter and a room temperature ^119m^Sn/CaSnO_3_ radioisotope source (Ritverc; ∼ 2.5 mCi) at a distance of 20 mm from the sample. The count rate was ∼ 11,000 counts/s in each channel, and all samples were measured for at least 12 days. Folding and calibration of the velocity axis was performed using the spectrum of a 25 μm-thick α-Fe foil (Ritverc) measured with a ^57^Co/Rh radioisotope source (Ritverc) as a reference. Isomer shifts are reported relative to natural abundance bulk SnO_2_ powder.

All spectra were binned by a factor of 5 before fitting; unbinned spectra are shown in Figures S29–S31, while binned spectra and corresponding fits are shown in Figures 4, S32. Spectra were fit to the sum of two or three Lorentzian functions, and all fit parameters are reported in Table S4. The spectrum of the 40% Sn as-synthesized material required three Lorentzian components, while only a marginal improvement in the fit was found upon adding a third component for the spectra of the 20% Sn as-synthesized material; fits of the spectra of both post-annealed materials converged to a solution with zero contribution from a third component. Although we cannot conclusively rule out the presence of the third component at ∼ 0.6 mm/s for the 20% Sn as-synthesized material, it is clearly not a major contributor to the spectrum, as demonstrated in Figure S33.

The peaks near 0 mm/s that are assigned to surface SnO_2_ show slight deviations from the expected isomer shift of exactly 0 mm/s, especially in the 40% Sn as-synthesized case (−0.21 mm/s). Marginal improvements in the fits were obtained when allowing all isomer shifts to vary as free parameters (vs fixing the shift of one component at 0 mm/s), and we choose to report these fit parameters because of the strong overlap between the peaks at ∼ 0 mm/s and ∼ 0.6 mm/s. Finally, we note that the linewidths of the peaks near 0 mm/s are narrower than the natural linewidth of 0.64 mm/s for the 23.88 keV nuclear transition in ^119^Sn. However, this is likely an artifact of the very small number of data points in the binned spectra that lie outside the noise floor in this region. Moreover, the natural linewidth falls within the error bars obtained for the fits.

### Transient Absorption Spectroscopy

Ultrafast pump–probe transmission measurements were conducted using a transient absorption spectrometer (Helios system, Ultrafast Systems) combined with an Astrella-F-1K femtosecond amplifier (800 nm and 1 kHz repetition rate). The fundamental 800 nm beam was split into probe and pump beams. The white-light probe was generated by focusing the fundamental beam into a sapphire crystal. Simultaneously, the remaining beam was directed to an optical parametric amplifier to produce a tunable pump beam. Samples in liquid form were placed on a moving sample holder to mitigate laser-induced degradation; this was achieved by continuous movement in an oval pattern over a 5 mm by 5 mm area. Both pump and probe beams traversed the sample, with the detector capturing data on every probe pulse to construct the absorption spectrum. To guarantee consistency, ten scans were collected for each transient absorption (TA) spectrum.

### Nonradiative Linewidth Calculations

In plasmonic nanoparticles, total homogeneous linewidth Γ is determined by fitting the dipolar extinction peak with a sum of Gaussian and Lorentzian functions, which helps account for peak asymmetry. This method works best for particles that do not exhibit higher-order resonances (e.g., quadrupoles), thus the values determined for the linewidths are limited to the particle radius at which a quadrupole is observed for each element.^32^

The total linewidth is determined by the combination of the radiative and nonradiative contributions^34,39,46^

1 $$\Gamma =\Gamma_{\mathrm{radiative}}+\Gamma_{\mathrm{non}‐\mathrm{radiative}}$$

where Γ is the linewidth of the LSPR based on *Q*_ext_, and Γ_nonradiative_ is determined from the dielectric function according to^34,35,39^

2 Γnon‐radiative=2Im{ε}(∂Re{ε}/∂ω)2+(∂Im{ε}/∂ω)2

where Γ_nonradiative_ is the linewidth associated with a dielectric function, Γ_radiative_ is determined from Γ_radiative_= Γ – Γ_nonradiative_. Equation 2 accounts for the bulk contributions to the linewidth derived from the dielectric function but does not account for surface scattering (Figure 5).^34,35^ This is excluded because surface interactions can be strongly dependent on the unique surface chemistry of a nanoparticle, which would differ significantly for the metals described herein.^33,39,47^

## Supplementary Material



## Notes

Additional methods, nanoparticle size characterization, X-ray characterization, and spectroscopy (PDF)
