# Supplementary material for: Annealing-Driven Phase Control Enables Plasmonic Tunability in Alloy Nanoparticles
Source: Chem Mater. 2025 Oct 21;37(21):8755–63. doi: 10.1021/acs.chemmater.5c01692 (PMC12613311; doi:10.1021/acs.chemmater.5c01692)
Supplement: Supplementary file 1 [file cm5c01692_si_001.pdf]

Supporting Information For:

Annealing-Driven Phase Control Enables Plasmonic Tunability in Alloy Nanoparticles

Noah L. Mason[1], Anthony J. Branco[1], Sunhao Liu[2], Maëlisse Trancart[1], Sangmin Jeong[1], Connor S. Sullivan[1], Sarah S. Dawes[1], Smita Chatterjee[1], Sophia Manukian[1], Dugan Hayes[3], Lina Quan[2], Michael B. Ross[*1]

[1]University of Massachusetts Lowell, Department of Chemistry, Lowell, MA 01854, USA

[2]University of North Carolina at Chapel Hill, Department of Chemistry, Chapel Hill, NC 27599, USA

[3]University of Rhode Island, Department of Chemistry, Kingston, RI 02881, USA

Supporting Tables and Figures

Table S1: Average size and polydispersity of Au-Sn nanoparticles synthesized at 60 °C before and after annealing at 80 °C for one hour (n=20).

Sample	Diameter (nm)	Coefficient of Variation (%)
Au Seeds	13.25	5.15
20% Sn As-Synthesized	13.42	5.43
20% Sn Annealed	13.53	5.69
40% Sn As-Synthesized	14.71	9.83
40% Sn Annealed	14.10	8.77

Table S2: Average Sn Composition (at.%) for 20% Sn-added Au-Sn As-Synthesized, 20% Sn-added Au-Sn Annealed for 1 hour, 40% Sn-added Au-Sn As-Synthesized, 40% Sn-added Au-Sn Annealed for 1 hour.

	20% Au-Sn As-Synthesized	20% Au-Sn Annealed	40% Au-Sn As-Synthesized	40% Au-Sn Annealed
STEM-EDS	7.73	2.26	36.23	8.29
ICP-OES	18.83	1.98	38.29	6.40
XPS	38.2	1.0	54.4	3.3

Table S3: ^{119}Sn Mössbauer Fit Parameters

Parameter	20% Sn As-Synthesized (mm/s)	20% Sn Annealed (mm/s)	40% Sn As-Synthesized (mm/s)	40% Sn Annealed (mm/s)
Isomer Shift 1	0.04±0.04	0.08±0.08	-0.21±0.08	0.09±0.06
Linewidth 1 (FWHM)	1.26±0.12	0.51±0.26	0.89±0.24	0.49±0.20
Amplitude 1	81%	21%	21%	11%
Isomer Shift 2	1.96±0.31	2.04±0.04	2.28±0.03	2.09±0.02
Linewidth 2 (FWHM)	1.88±0.99	0.77±0.13	1.11±0.10	0.85±0.06
Amplitude 2	19%	79%	34%	89%
Isomer Shift 3	n/a	n/a	0.51±0.11	n/a
Linewidth 3 (FWHM)	n/a	n/a	1.34±0.26	n/a
Amplitude 3	n/a	n/a	45%	n/a

Table S4: Electron-Phonon Time Constants Measured at (5.28×10^{-6} μJ•cm^{-1}).

Sample	Phonon-Scattering Time Constant (ps)
Au Seeds	3.79 ps±1.3 fs
10% Sn Added	3.95 ps±1.7 fs
20% Sn Added	3.46 ps±2.9 fs
30% Sn Added	3.30 ps±0.49 fs
40% Sn Added	2.88 ps ±0.66 fs

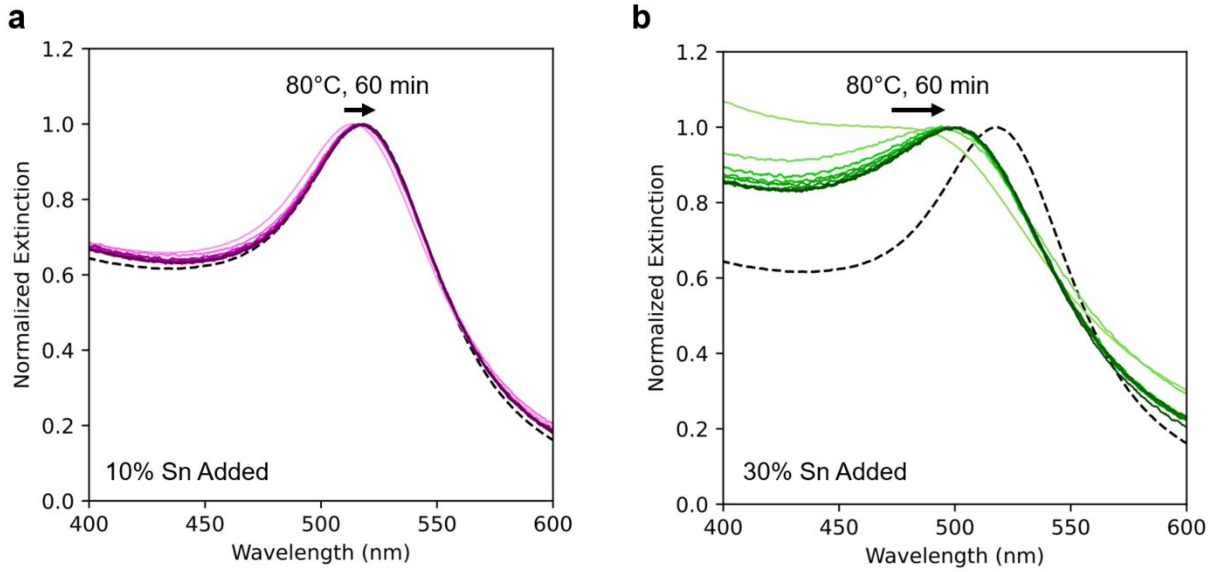

Figure S1. Normalized extinction spectra of (a) 10% and (b) 30% Sn-added Au-Sn nanoparticles taken at 0, 2.5, 5.0, 7.5, 10, 15, 20, 30, and 60 minutes while annealing at 80 °C.

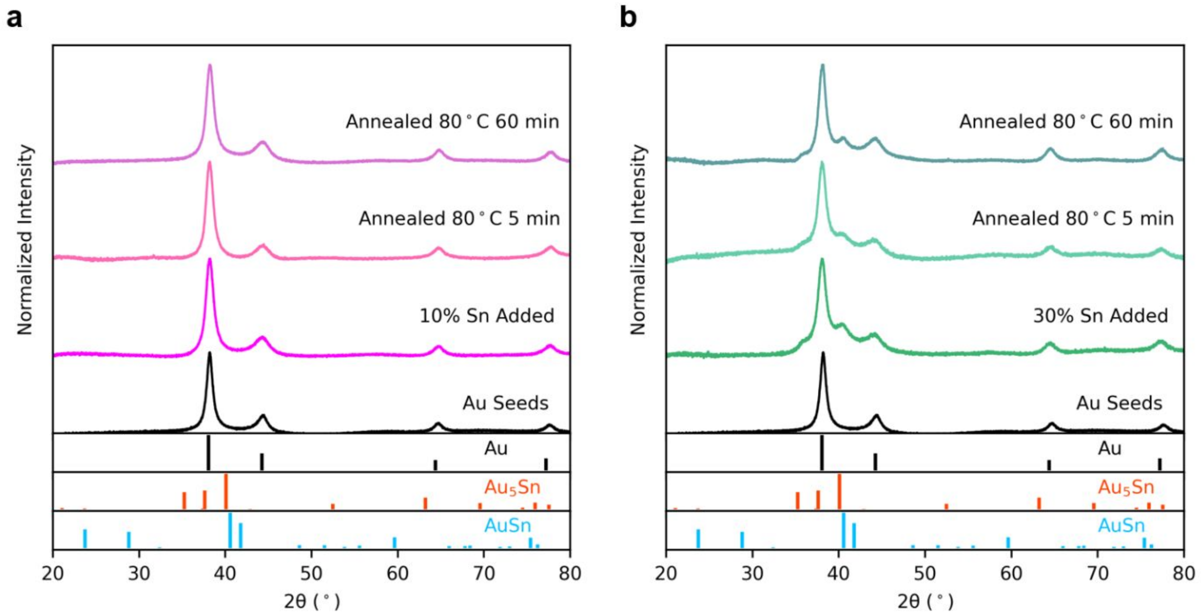

Figure S2. Waterfall XRD plots for (a) 10% (b) 30% Sn-added Au-Sn nanoparticles synthesized at 60 °C while annealing at 80 °C for one hour.

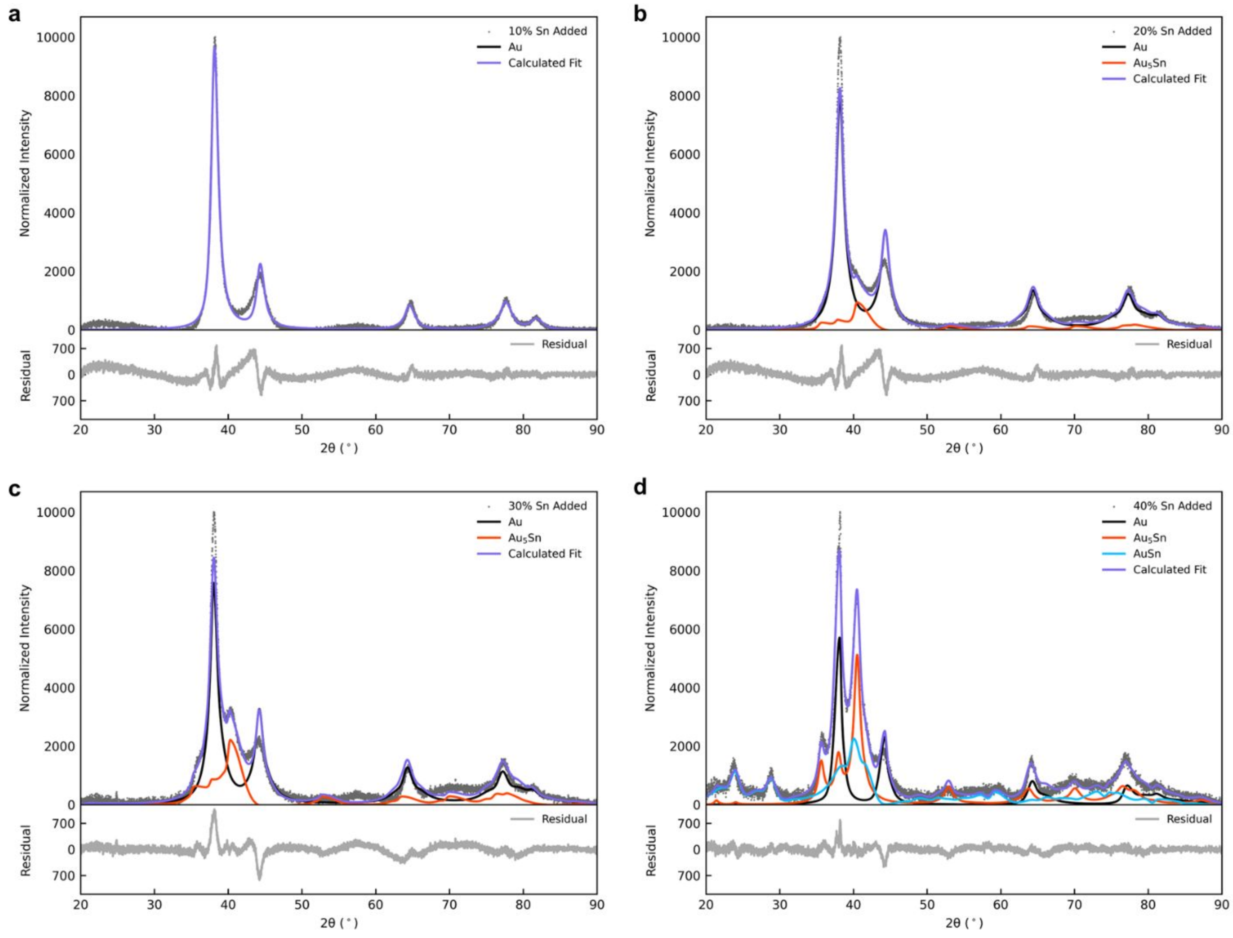

Figure S3. Rietveld refinement fits for (a) 10% (b) 20% (c) 30% and (d) 40% Sn-added Au-Sn nanoparticles as synthesized at 60 °C.

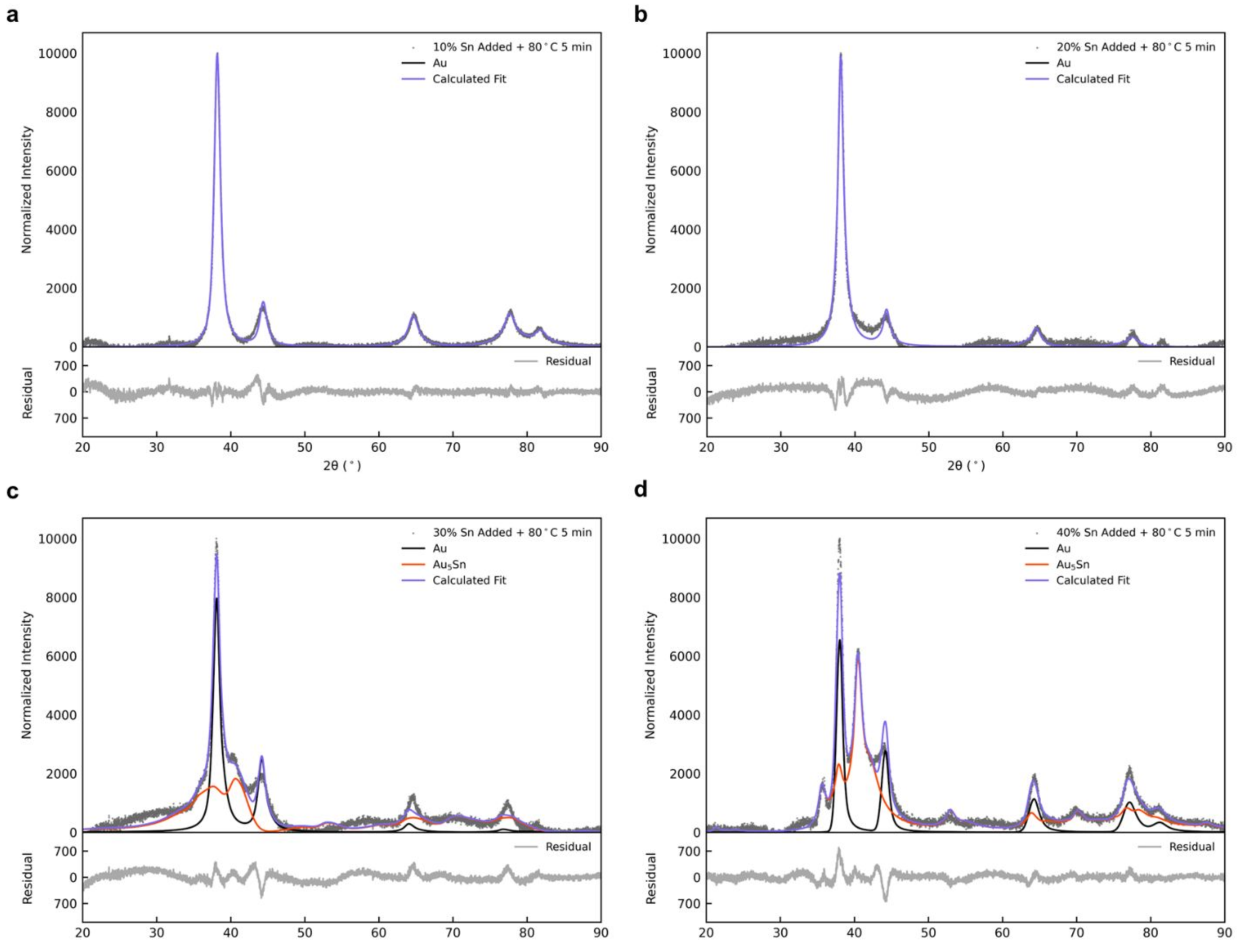

Figure S4. Rietveld refinement fits for (a) 10% (b) 20% (c) 30% and (d) 40% Sn-added Au-Sn nanoparticles synthesized at 60 °C and annealed at 80 °C for five minutes.

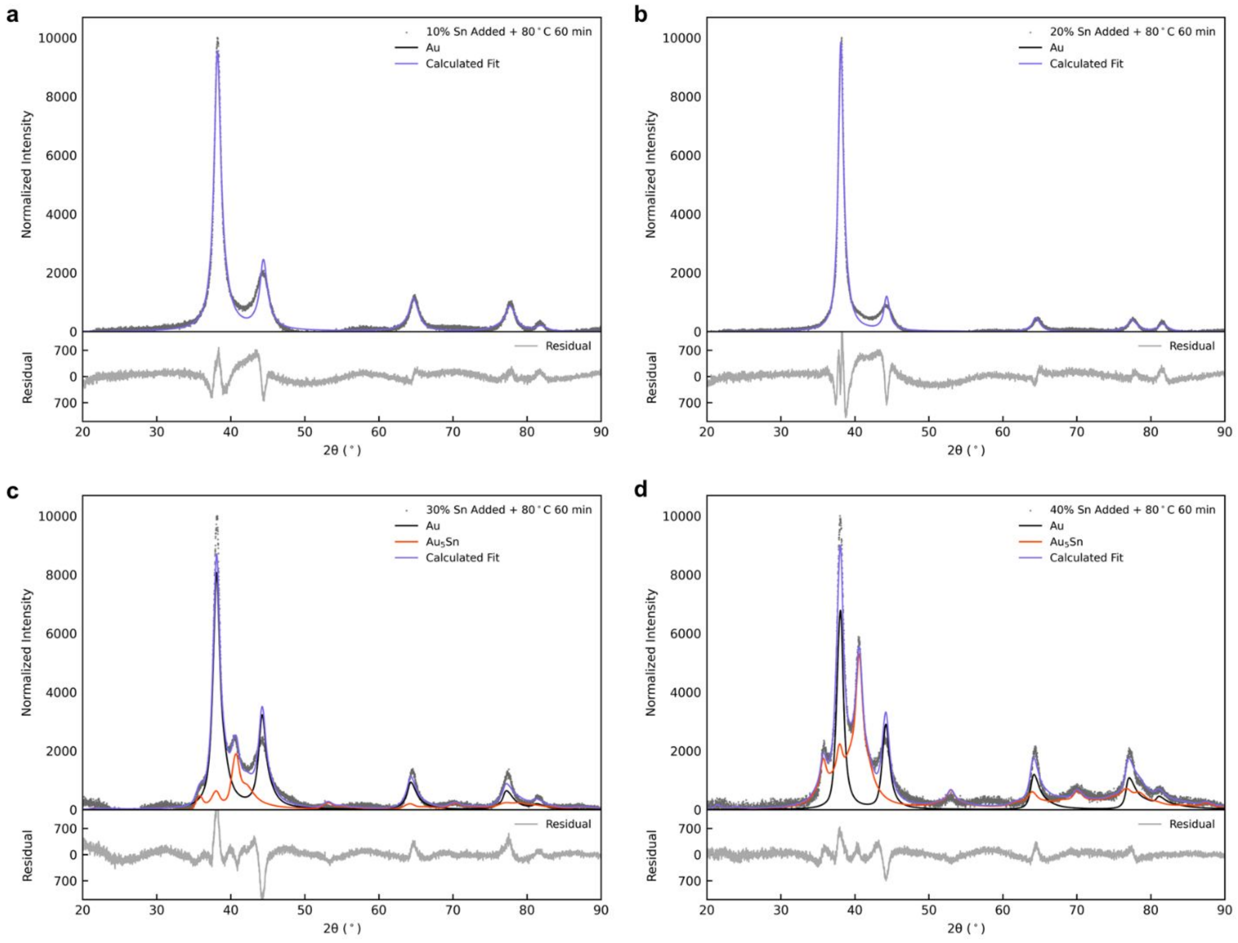

Figure S5. Rietveld refinement fits for (a) 10% (b) 20% (c) 30% and (d) 40% Sn-added Au-Sn nanoparticles synthesized at 60 °C and annealed at 80 °C for one hour.

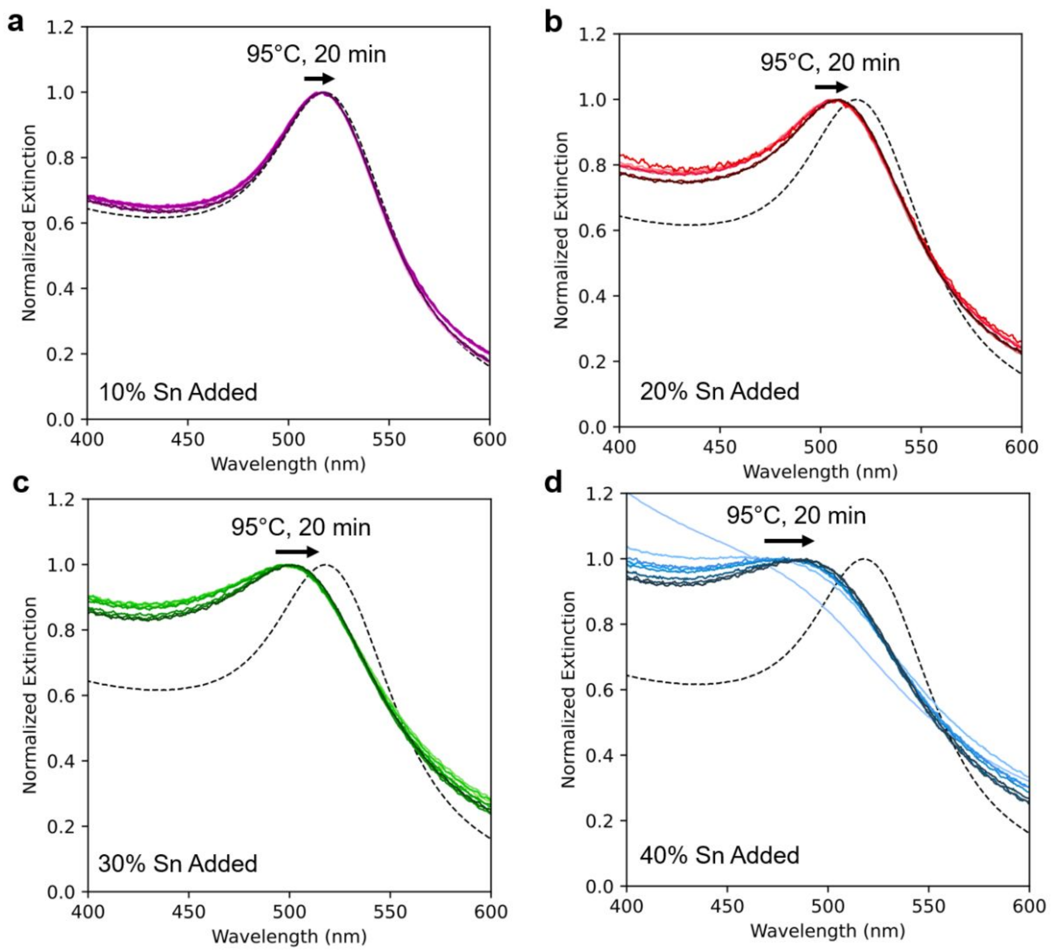

Figure S6. Normalized extinction spectra of (a) 10%, (b) 20%, (c) 30%, and (d) 40% Sn-added Au-Sn nanoparticles synthesized at 60 °C taken at 0, 2, 4, 6, 8, 10, 15, and 20 minutes while annealing at 95 °C.

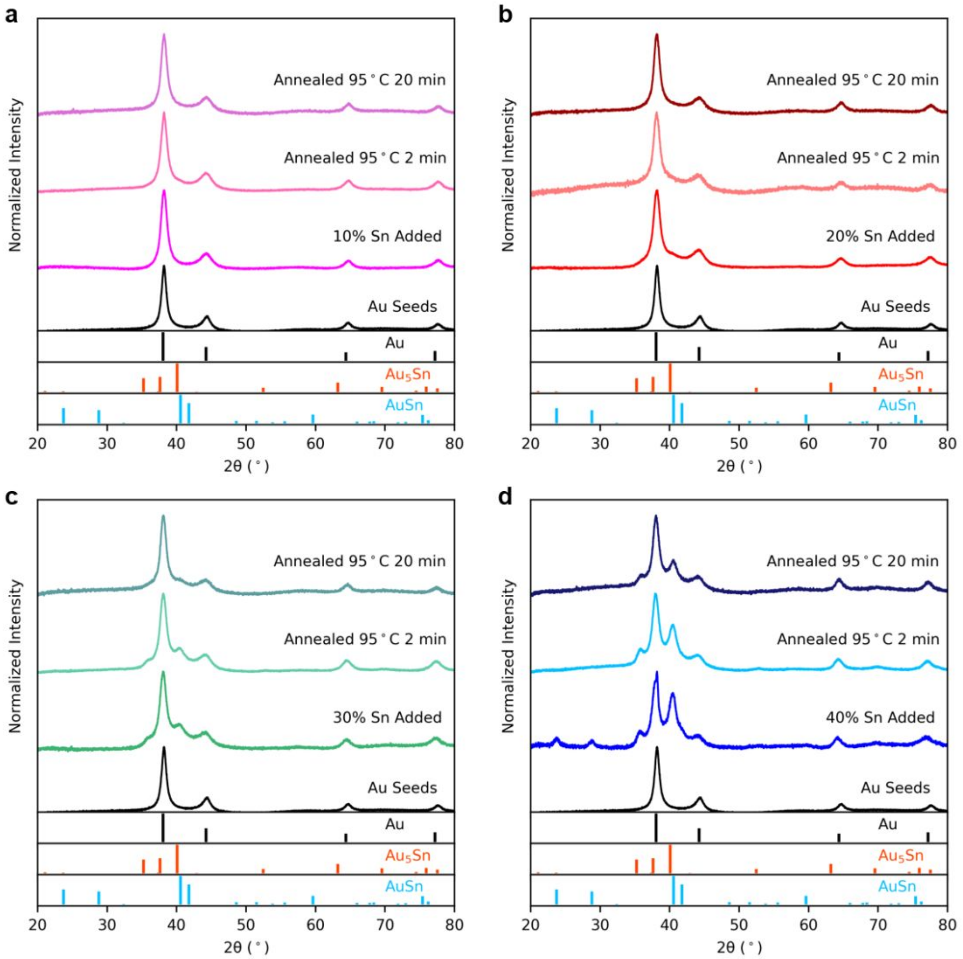

Figure S7. Waterfall XRD plots for (a) 10%, (b) 20%, (c) 30%, and (d) 40% added Au-Sn nanoparticles synthesized at 60 °C while annealing at 95 °C for 20 minutes.

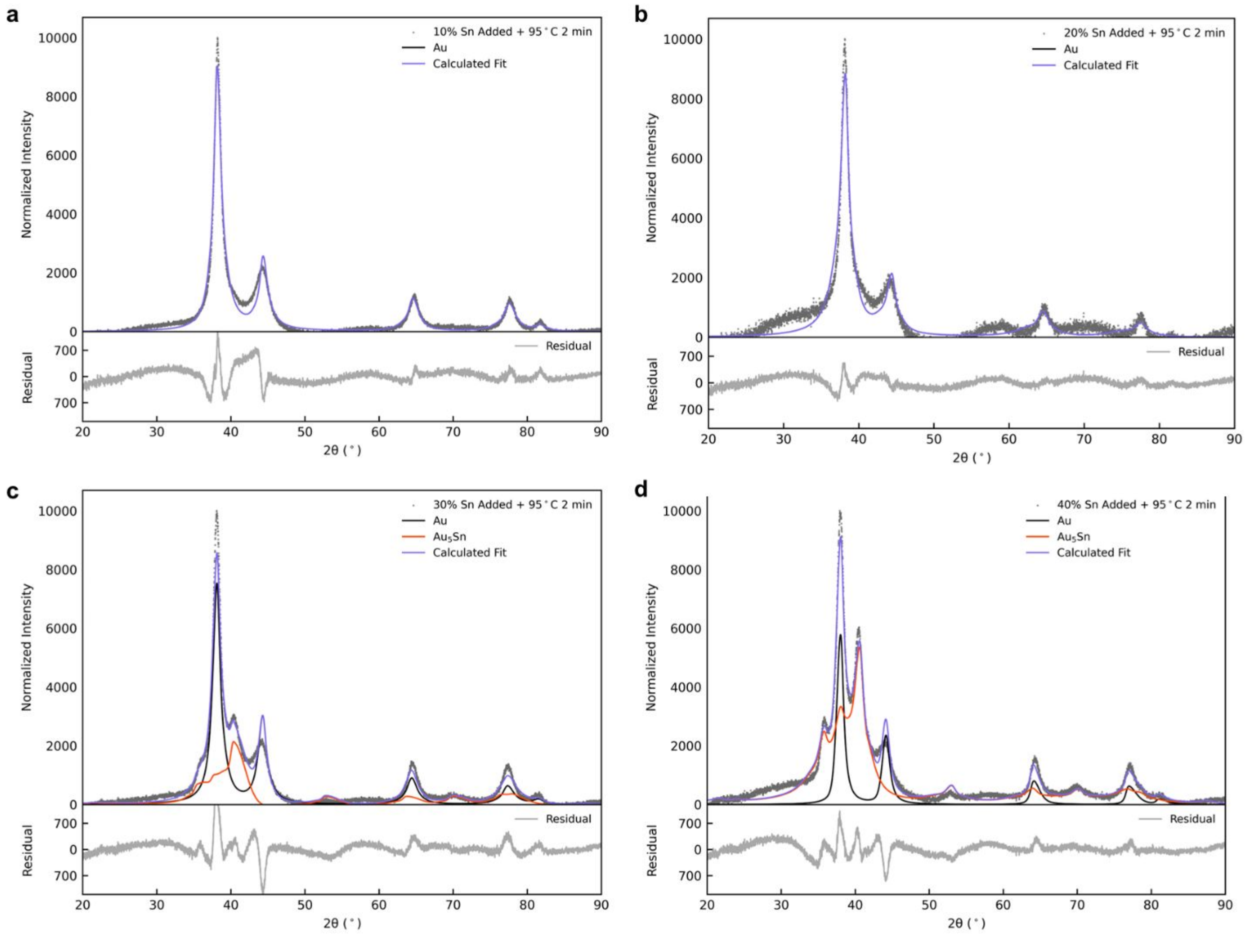

Figure S8. Rietveld refinement fits for (a) 10% (b) 20% (c) 30% and (d) 40% Sn-added Au-Sn nanoparticles synthesized at 60 °C and annealed at 95 °C for two minutes.

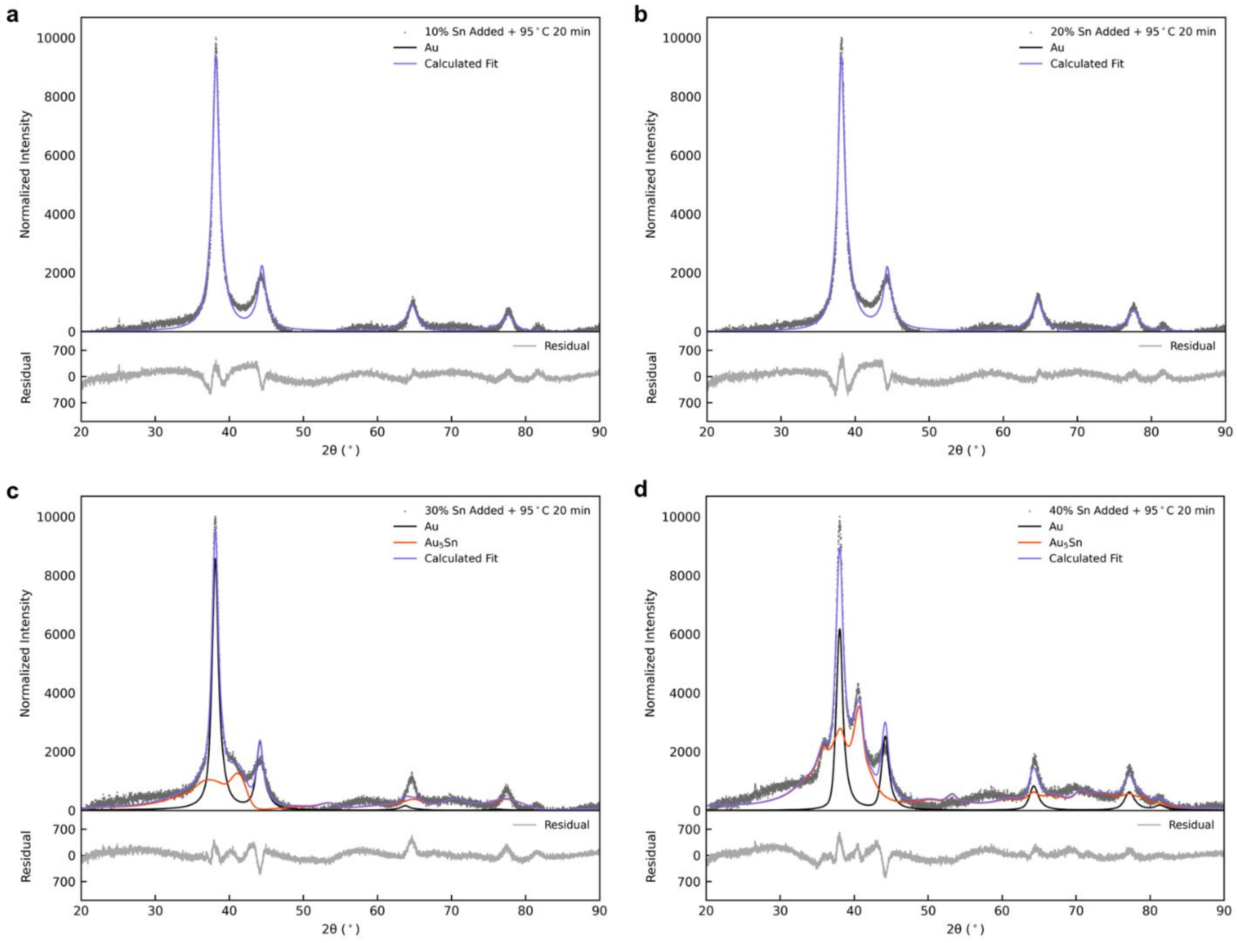

Figure S9. Rietveld refinement fits for (a) 10% (b) 20% (c) 30% and (d) 40% Sn-added Au-Sn nanoparticles synthesized at 60 °C and annealed at 95 °C for 20 minutes.

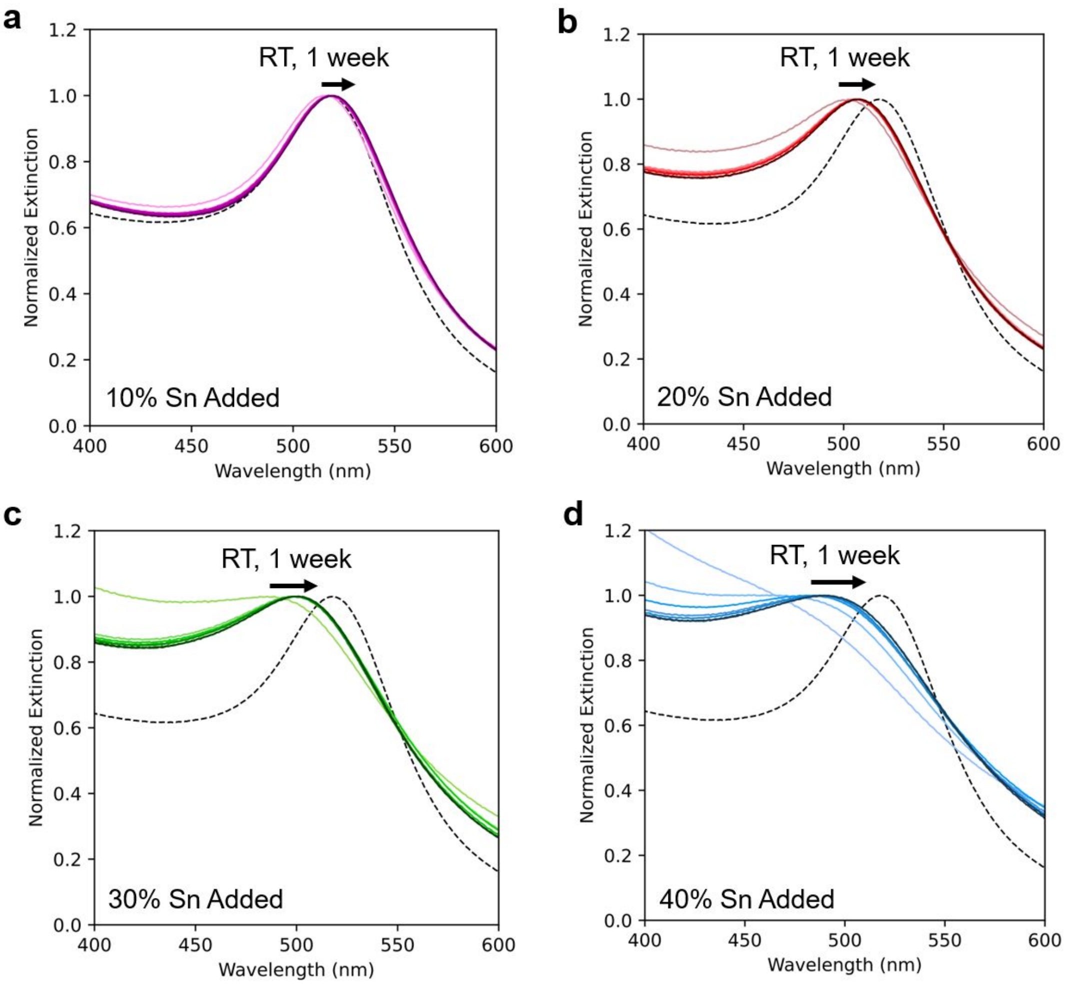

Figure S10. Normalized extinction spectra of (a) 10%, (b) 20%, (c) 30%, and (d) 40% Sn-added Au-Sn nanoparticles synthesized at 60 °C taken at 0, 12, 24, 48, 72, 96, and 168 hours (1 week) while annealing at room temperature.

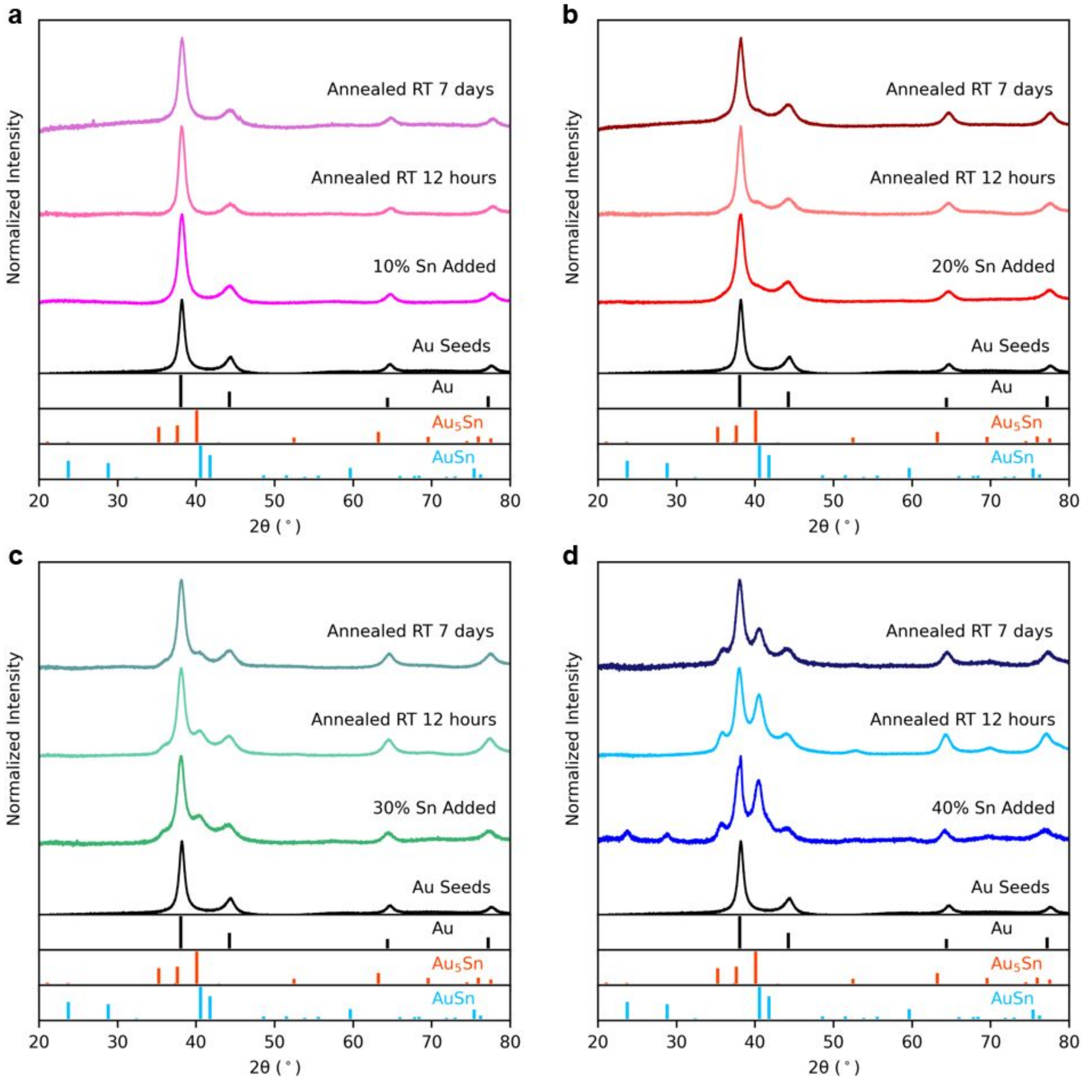

Figure S11. Waterfall XRD plots for (a) 10%, (b) 20%, (c) 30%, and (d) 40% added Au-Sn nanoparticles synthesized at 60 °C while annealing at room temperature for one week.

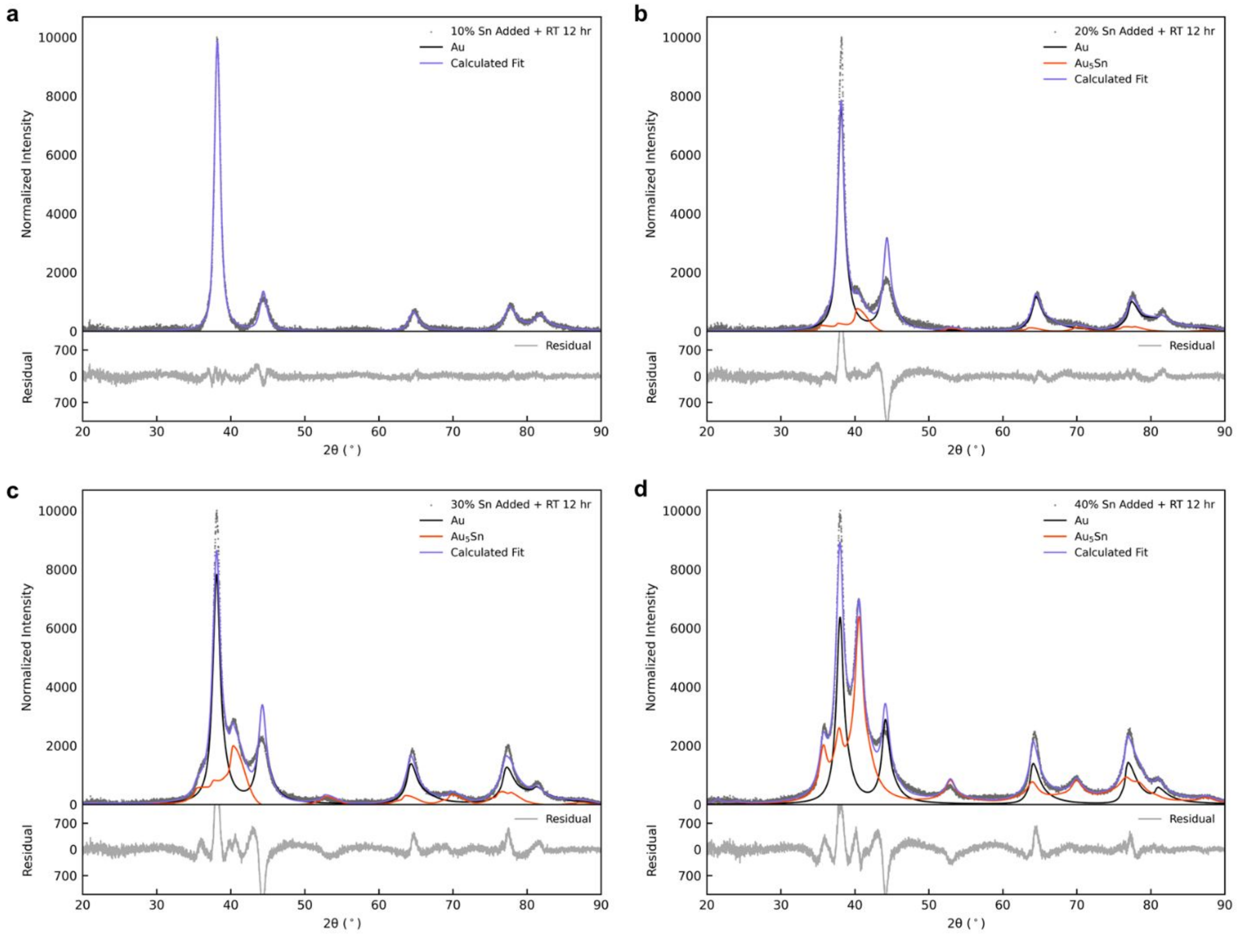

Figure S12. Rietveld refinement fits for (a) 10% (b) 20% (c) 30% and (d) 40% Sn-added Au-Sn nanoparticles synthesized at 60 °C and annealed at room temperature for 12 hours.

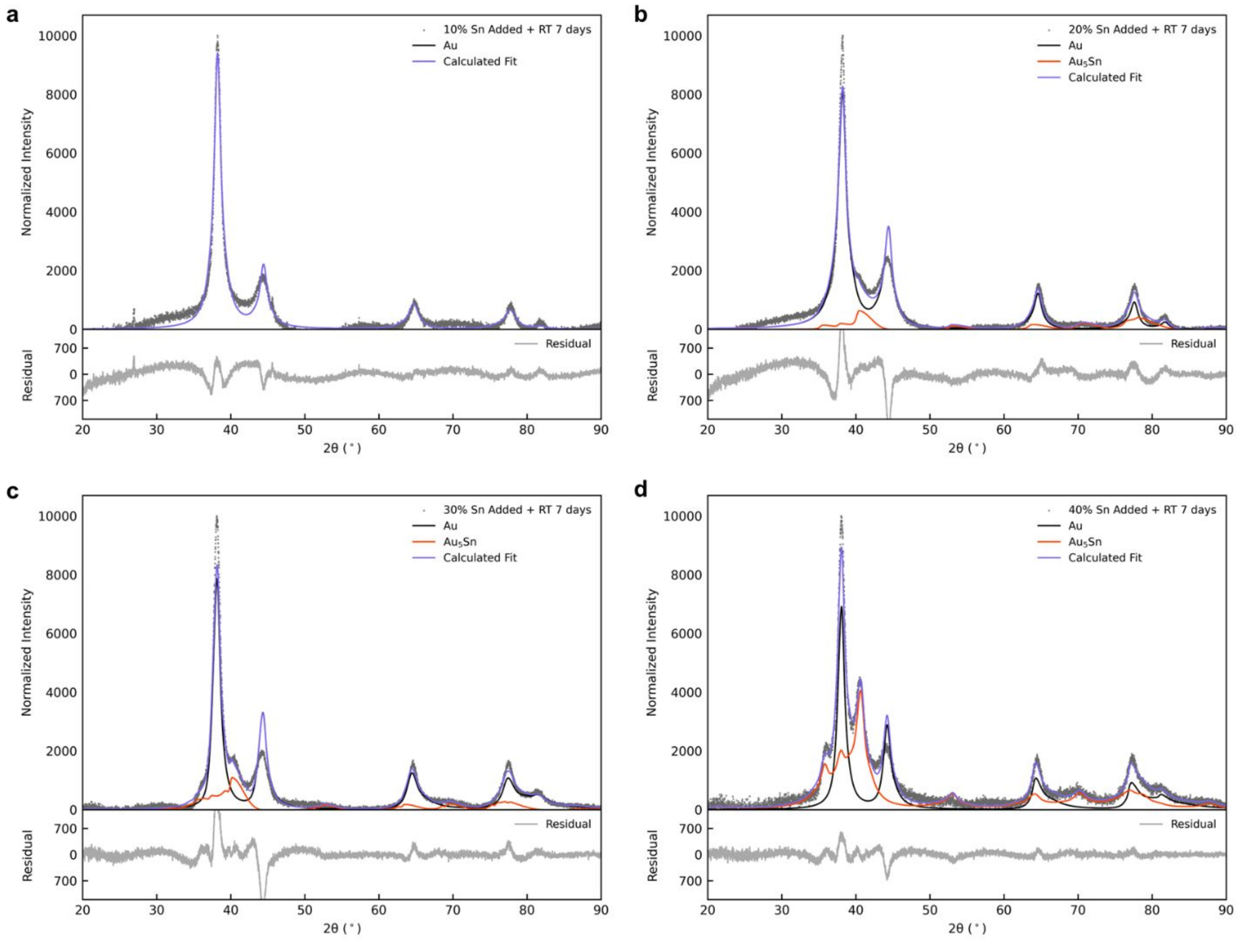

Figure S13. Rietveld refinement fits for (a) 10% (b) 20% (c) 30% and (d) 40% Sn-added Au-Sn nanoparticles synthesized at 60 °C and annealed at room temperature for one week.

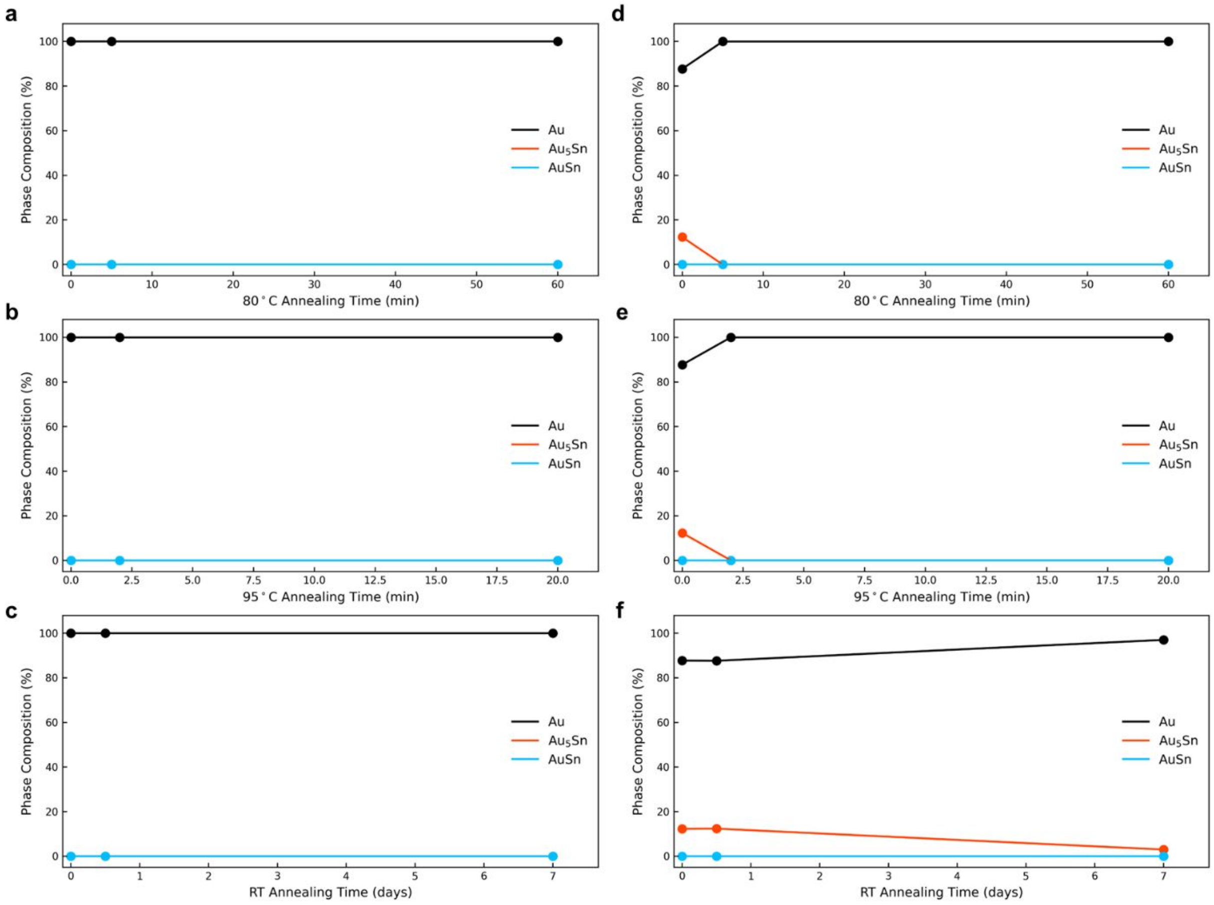

Figure S14. Individual percent intermetallic phase content from Rietveld refinements plotted as a function of annealing time at (a/d) 80 °C, (b/e) 95 °C, and (c/f) room temperature for (a-c) 10% and (d-f) 20% Sn-added Au-Sn nanoparticles.

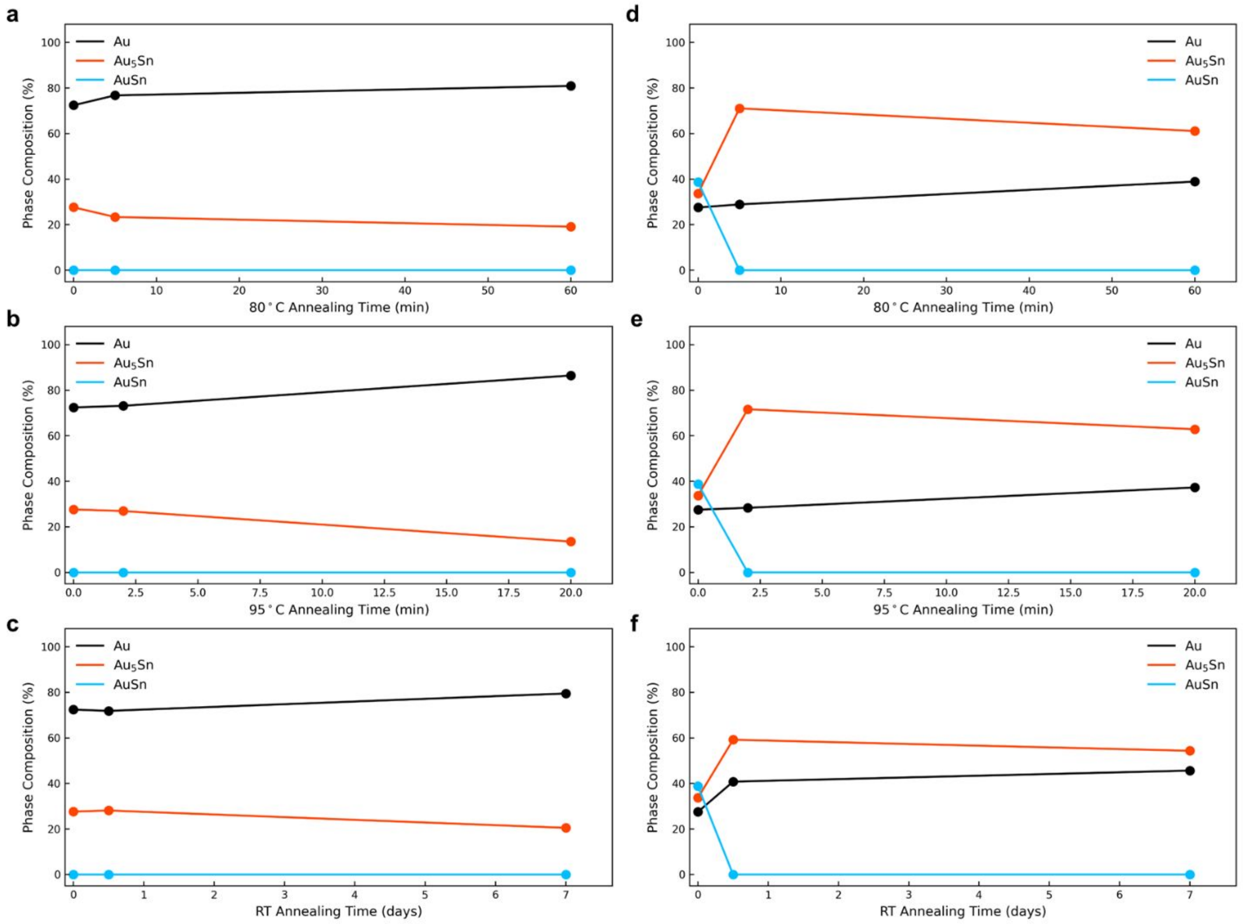

Figure S15. Individual percent intermetallic phase content from Rietveld refinements plotted as a function of annealing time at (a/d) 80 °C, (b/e) 95 °C, and (c/f) room temperature for (a-c) 30% and (d-f) 40% Sn-added Au-Sn nanoparticles.

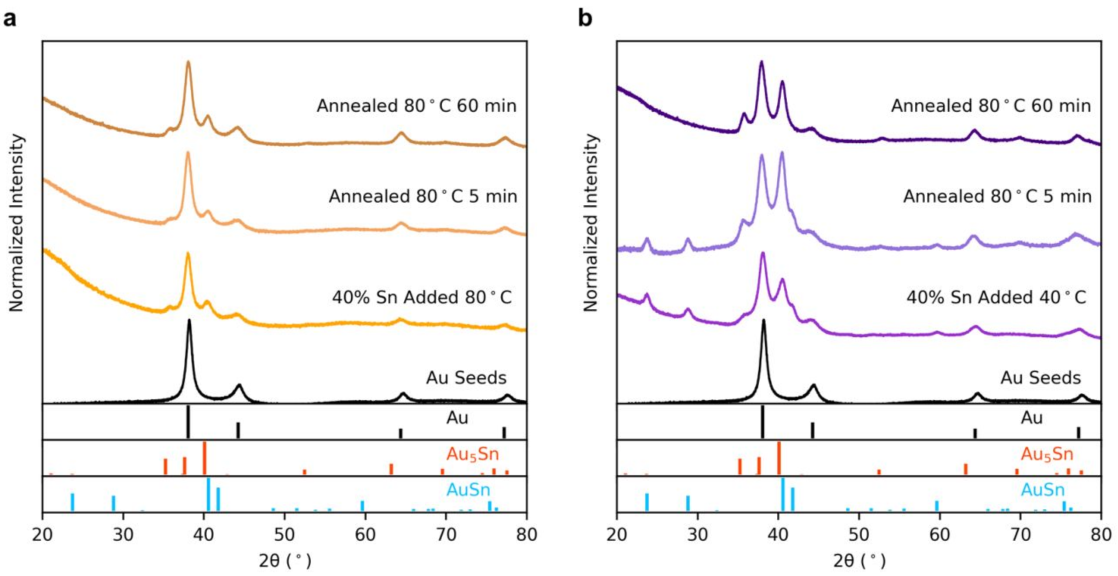

Figure S16. Waterfall XRD plots for 40% Sn-added Au-Sn nanoparticles synthesized at (a) 80 °C and (b) 40°C throughout annealing at 80 °C.

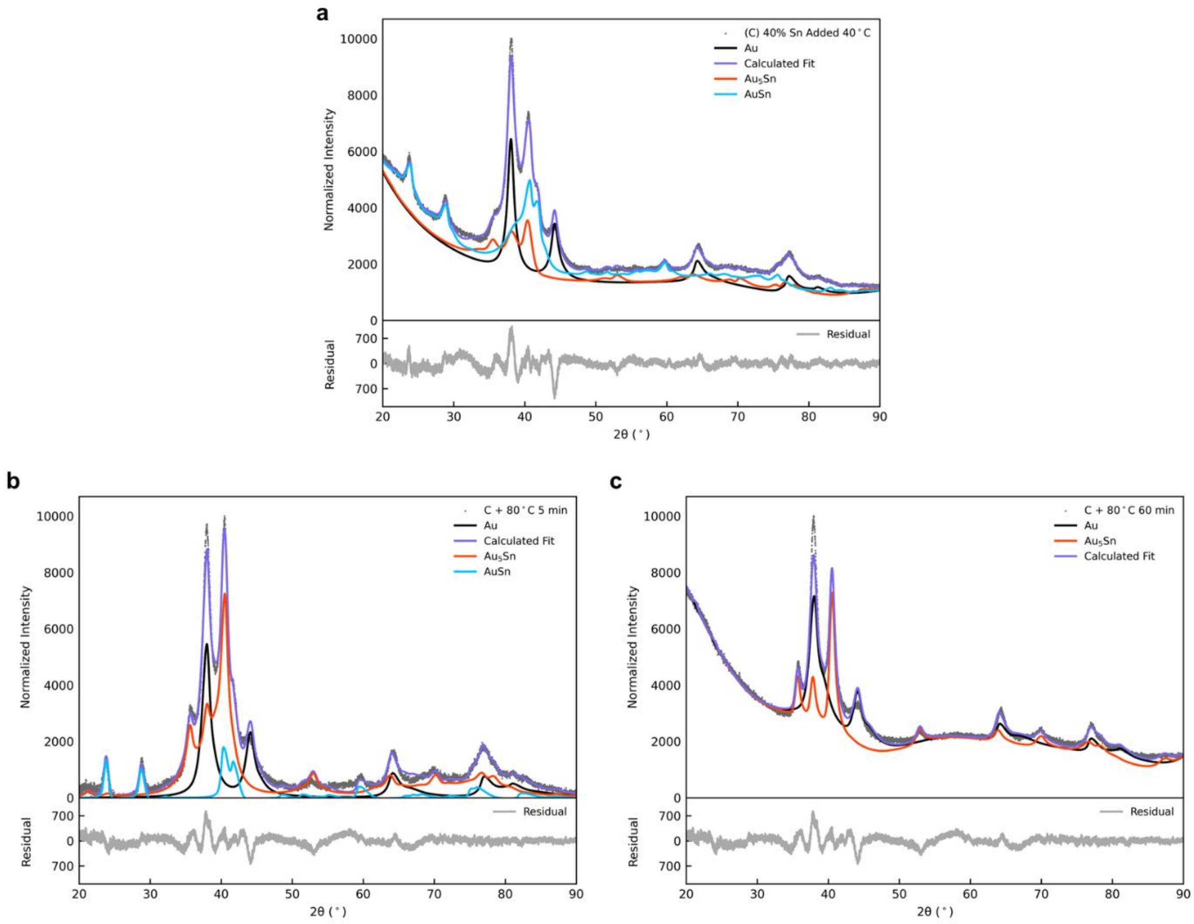

Figure S17. Rietveld refinement fits for 40% Sn-added Au-Sn nanoparticles (a) synthesized at 40 °C and annealed at 80 °C for (b) five minutes and (c) one hour.

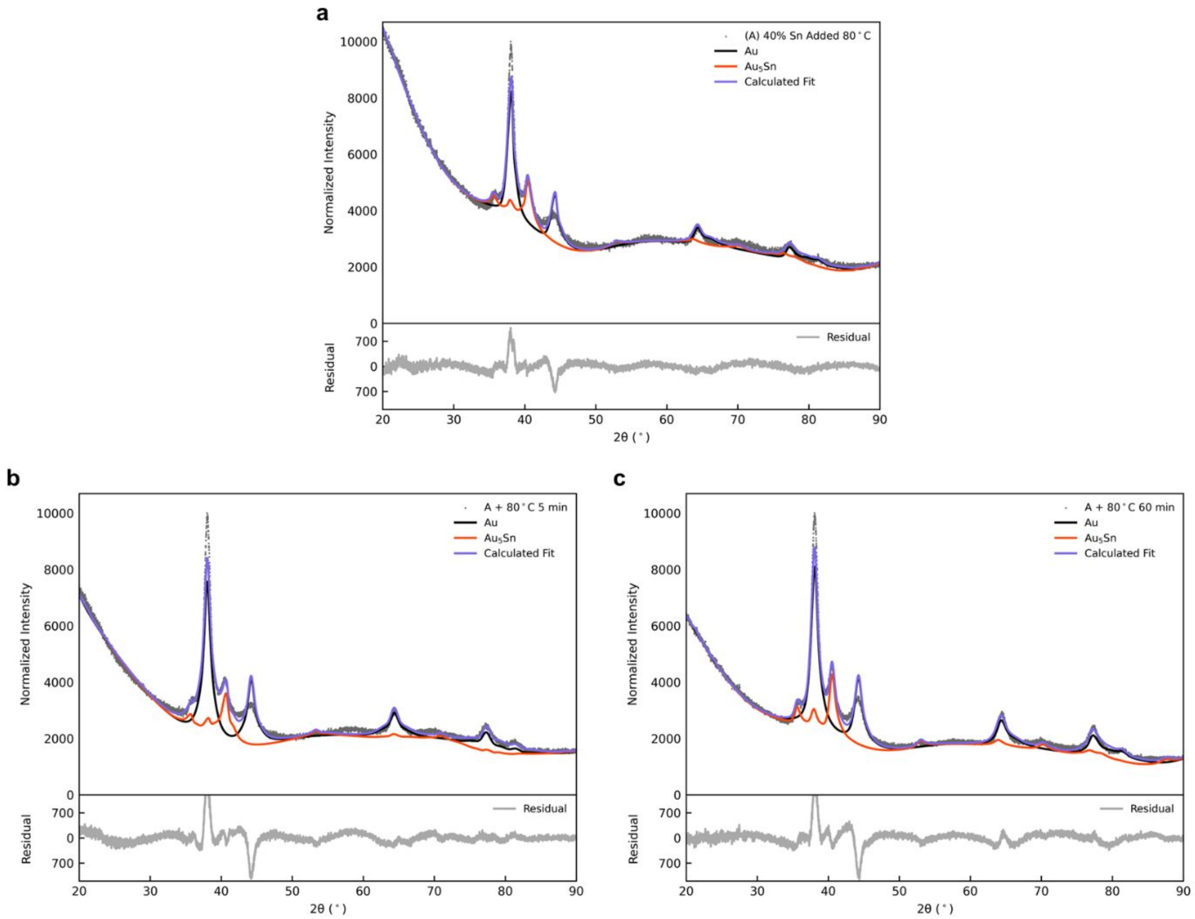

Figure S18. Rietveld refinement fits for 40% Sn-added Au-Sn nanoparticles (a) synthesized at 80 °C and annealed at 80 °C for (b) five minutes and (c) one hour.

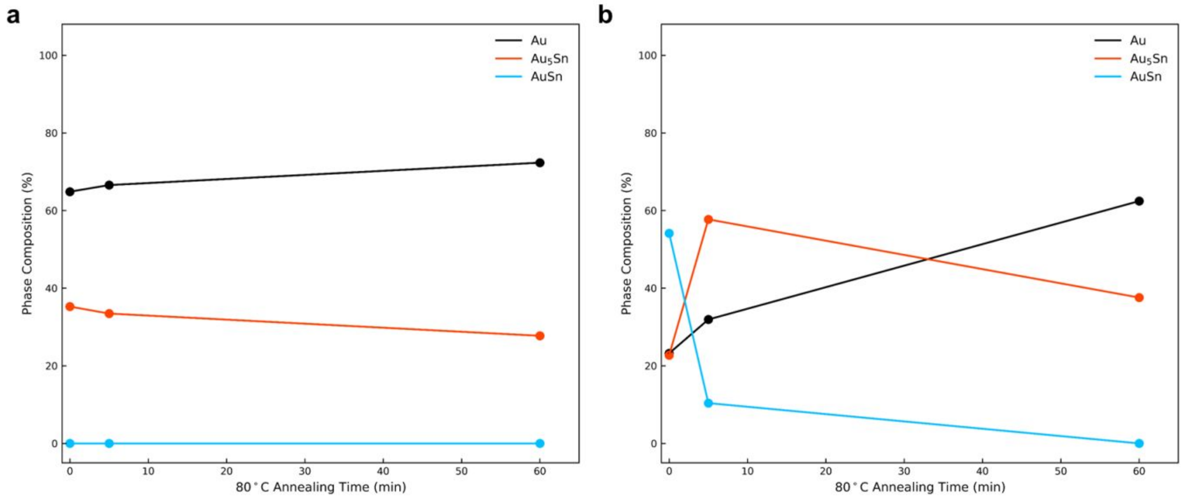

Figure S19. Individual percent intermetallic phase content from Rietveld refinements plotted as a function of annealing time at 80 °C for 40% Sn-added Au-Sn nanoparticles synthesized at (a) 80 °C and (b) 40 °C.

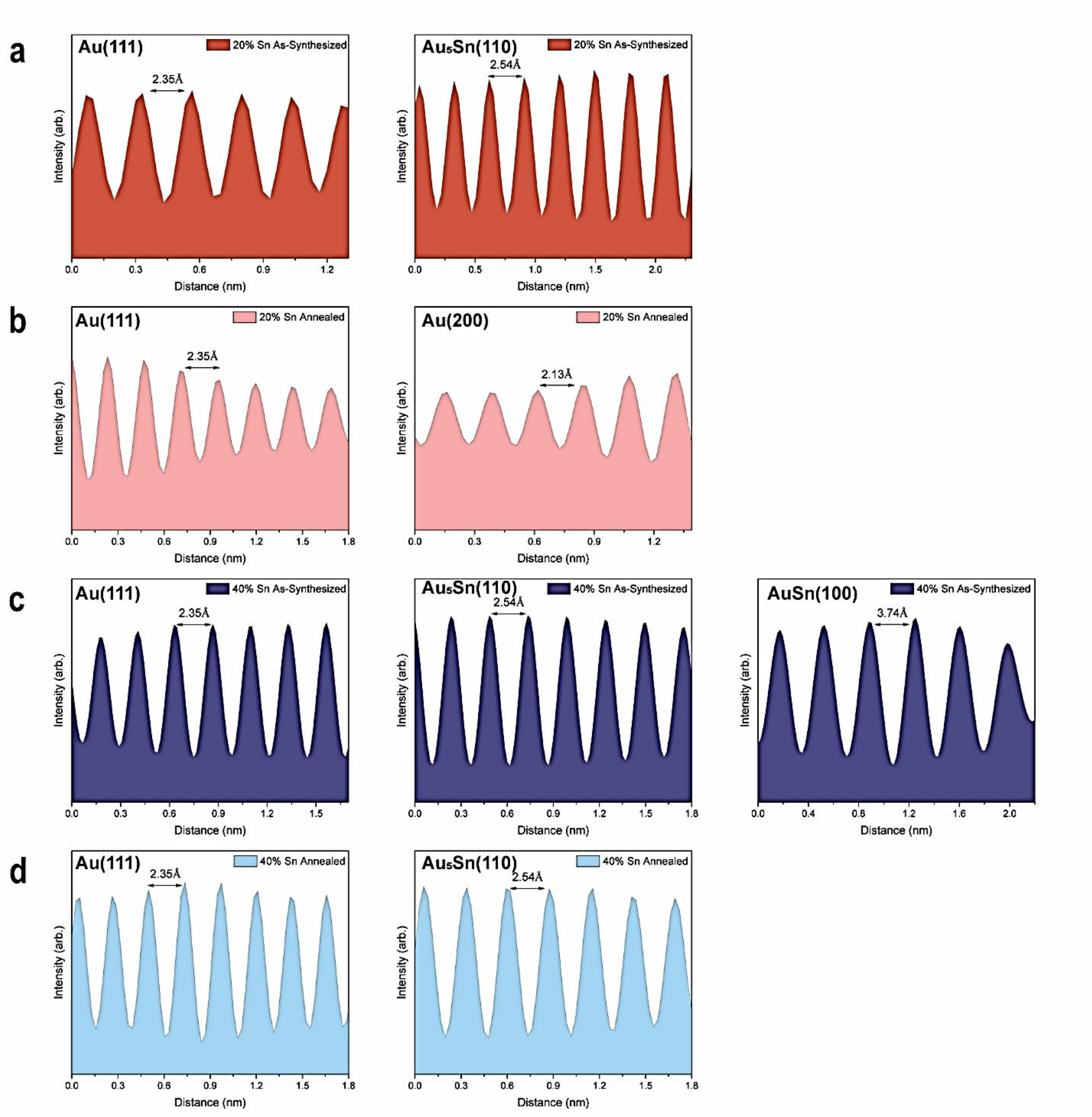

Figure S20. Intensity distributions from fast Fourier transforms (FFT) between lattice planes as observed in HRTEM imaging. The average calculated *d*-spacing is subsequently used to identify coexisting phases.

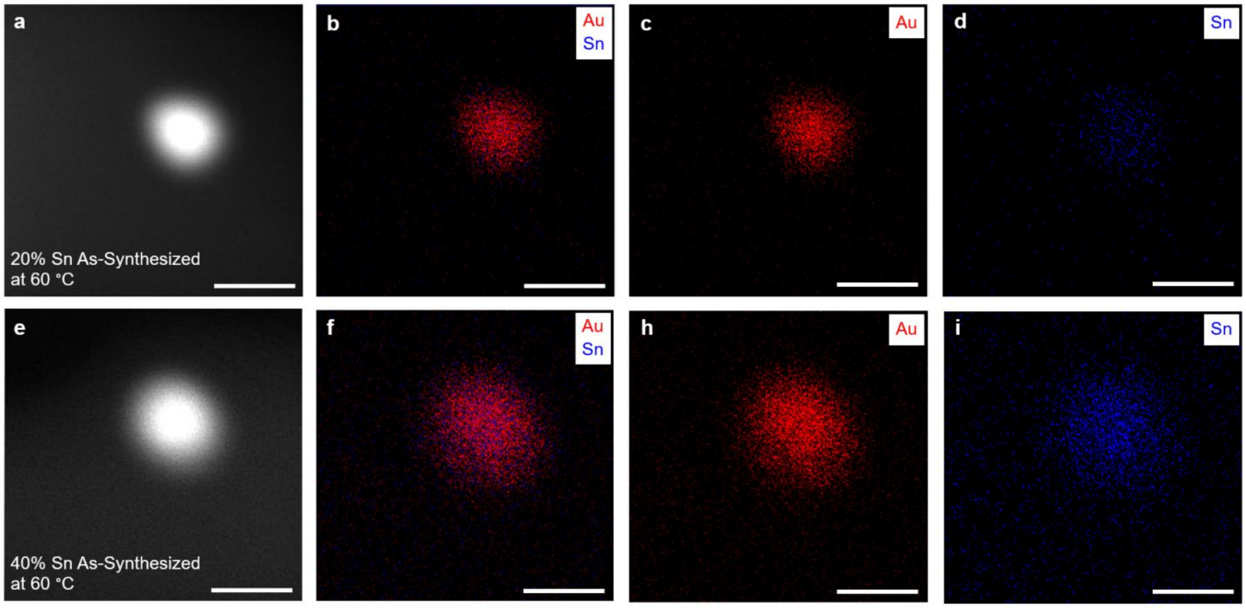

Figure S21. High-resolution DF-STEM EDX map of (a-d) 20% Au-Sn nanoparticles and (e-i) 40% Au-Sn nanoparticles synthesized at 60 °C. Scale bars are 20 nm.

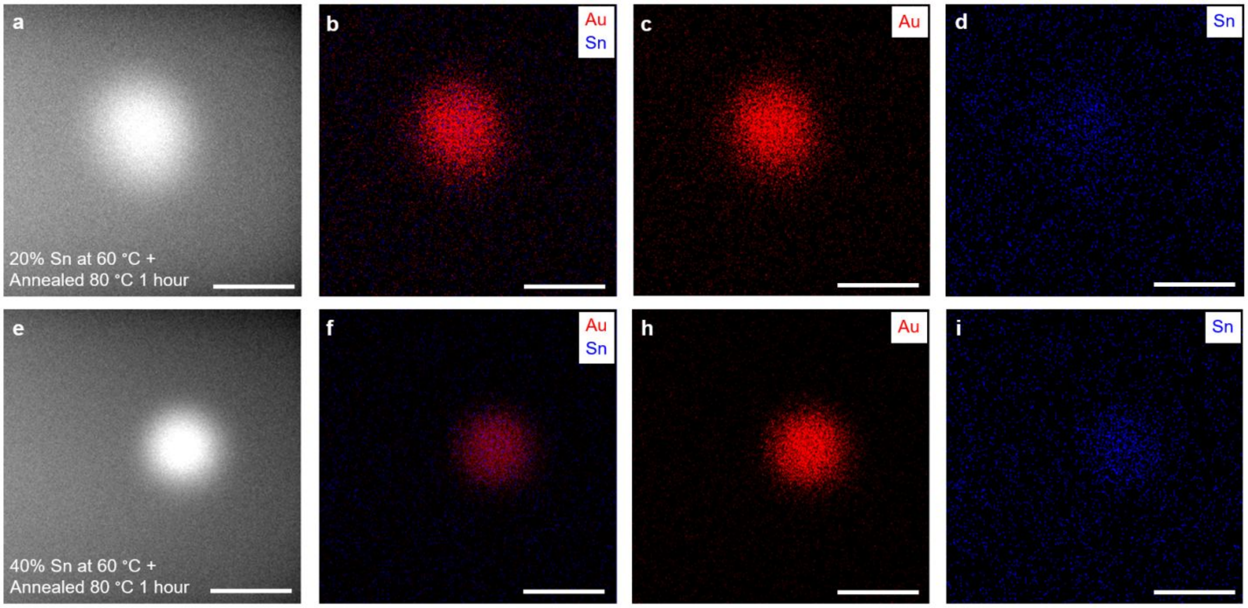

Figure S22. High-resolution DF-STEM EDX map of (a-d) 20% Au-Sn nanoparticles and (e-i) 40% Au-Sn nanoparticles synthesized at 60 °C and annealed at 80 °C for one hour. Scale bars are 20 nm.

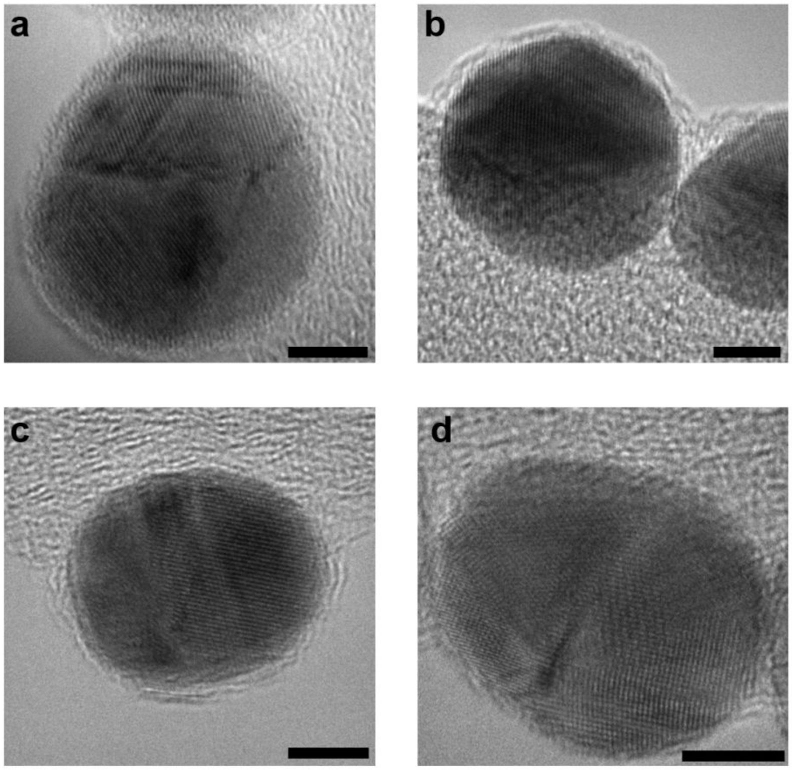

Figure S23. High-resolution transmission electron microscopy of 40% Sn-added Au-Sn nanoparticles synthesized at 40 °C (a) before and (b) after annealing and synthesized at 80 °C (a) before and (b) after annealing at 80 °C for one hour. All scale bars are 5 nm.

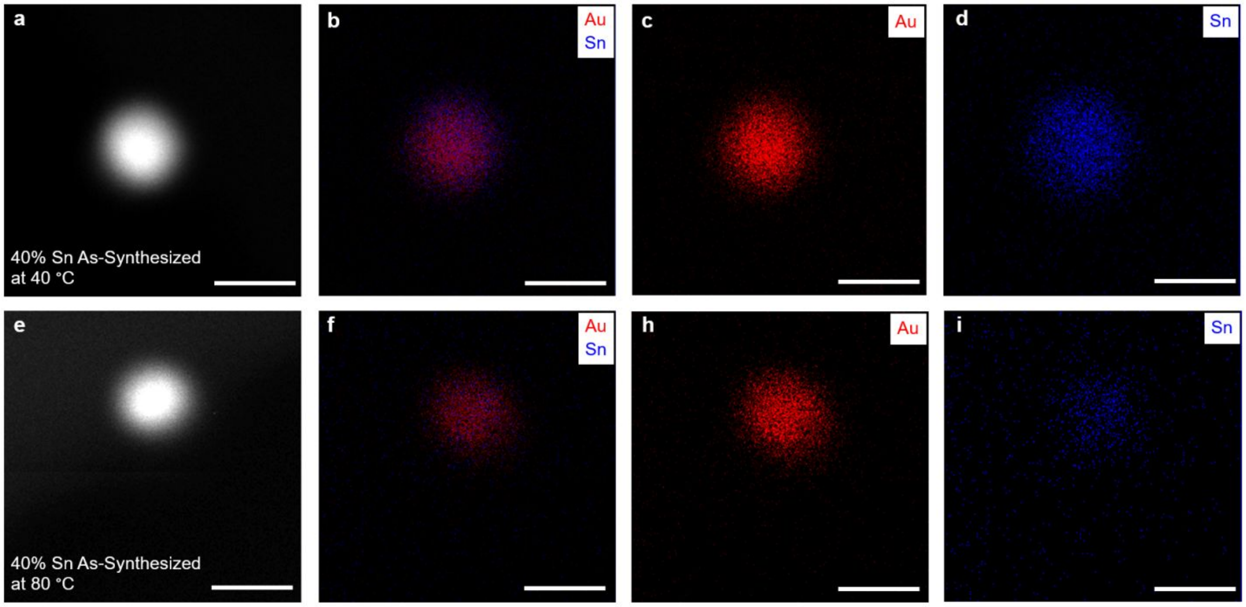

Figure S24. High-resolution DF-STEM EDX maps of (a-d) 40% Sn-added Au-Sn nanoparticles synthesized at 40 °C and (e-i) 40% Sn-added Au-Sn nanoparticles synthesized at 80 °C. Scale bars are 20 nm.

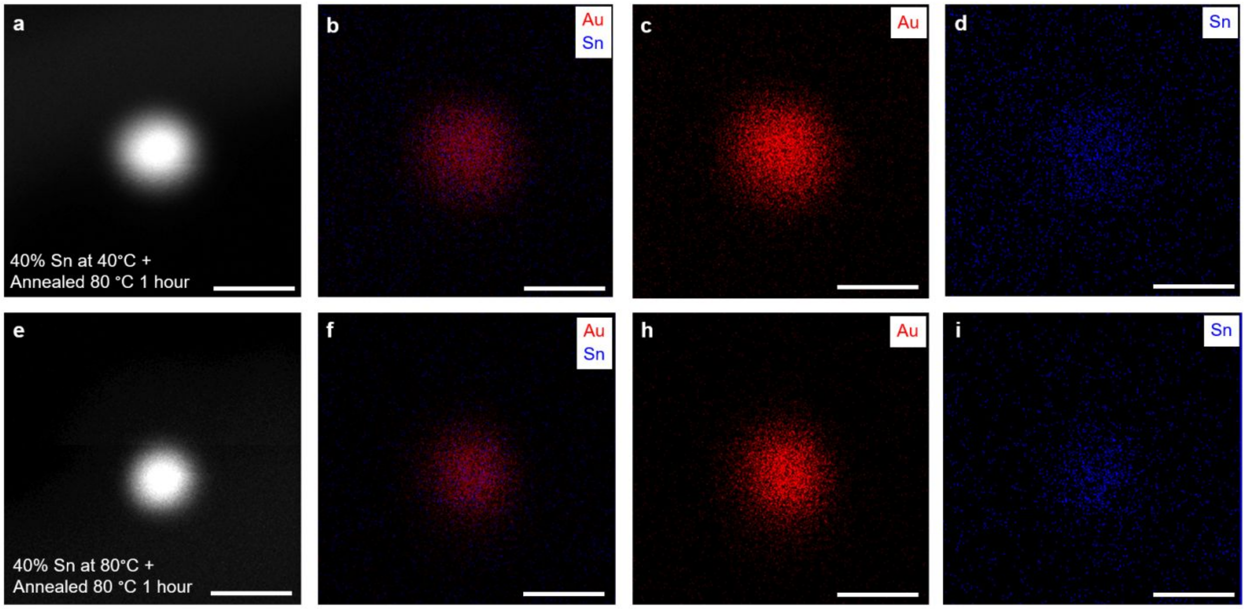

Figure S25. High-resolution DF-STEM EDX maps of (a-d) 40% Sn-added Au-Sn nanoparticles synthesized at 40 °C and (e-i) 40% Sn-added Au-Sn nanoparticles synthesized at 80 °C after annealing at 80 °C for one hour. Scale bars are 20 nm.

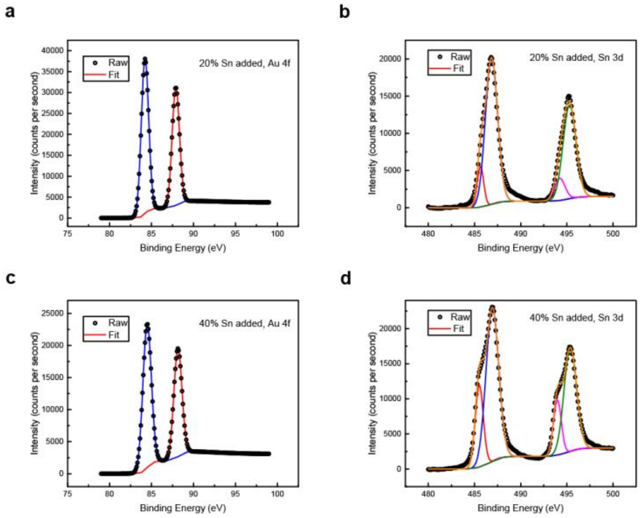

Figure S26. X-ray photoelectron spectra (a) 20% Sn added Au 4f, (b) 20% Sn added Sn 3d, (c) 40% Sn added Au 4f, and 40% Sn added Sn 3d nanoparticles synthesized at 60 °C.

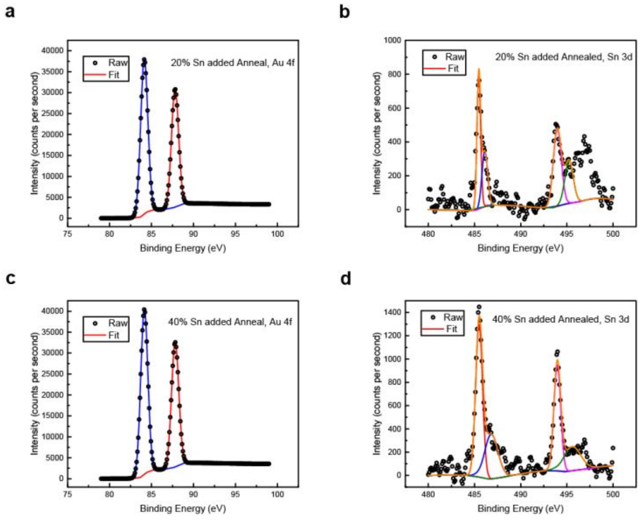

Figure S27. X-ray photoelectron spectra (a) 20% Sn added Au 4f, (b) 20% Sn added Sn 3d, (c) 40% Sn added Au 4f, and 40% Sn added Sn 3d nanoparticles synthesized at 60 °C and annealed at 80 °C for 1 hour.

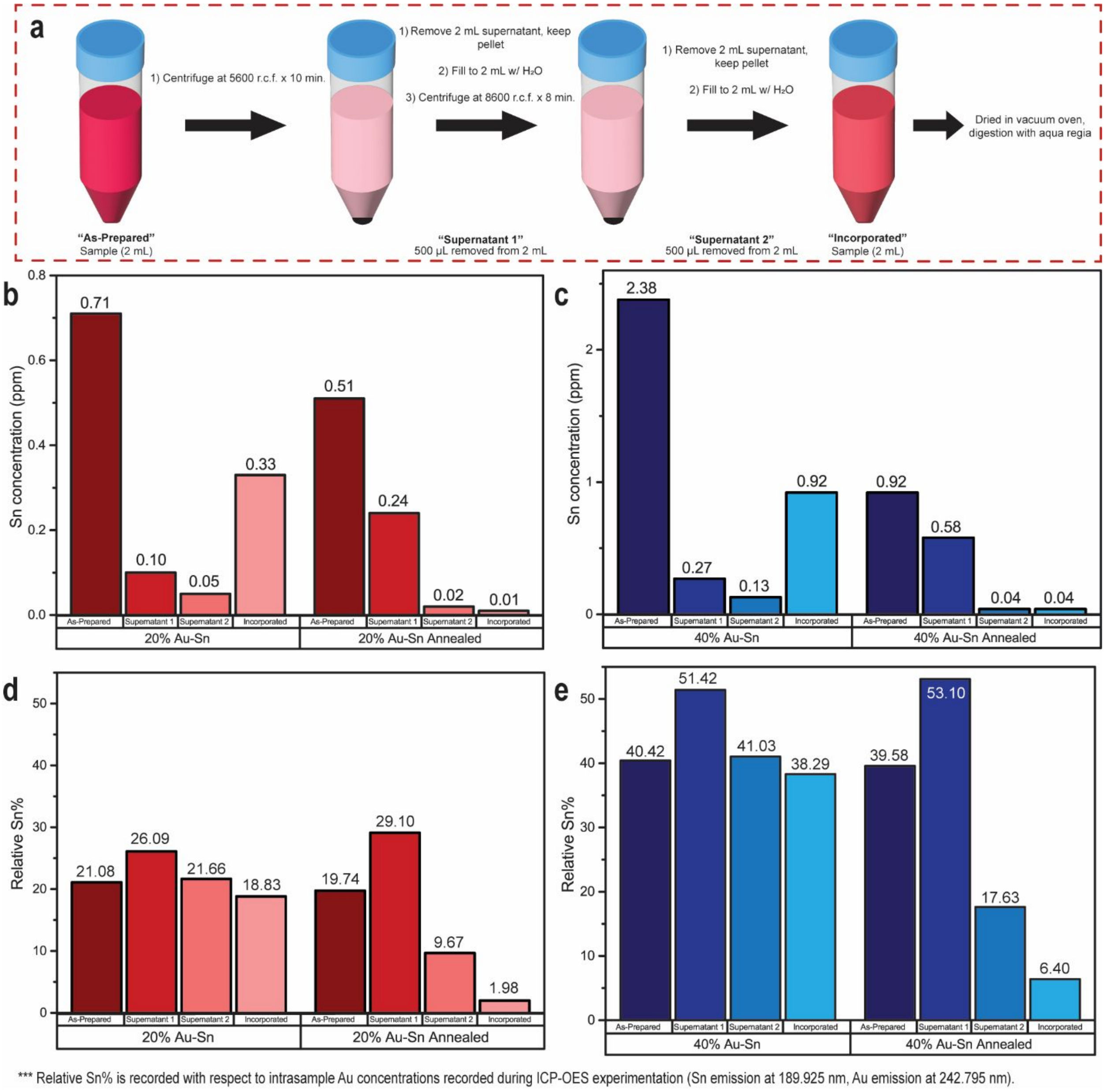

Figure S28. Inductively coupled plasma optical emission spectroscopy (ICP-OES) results for 20% Sn-added Au-Sn nanoparticles, 40% Sn-added Au-Sn nanoparticles, 20% Sn-added annealed for 1hr, and 20% Sn-added annealed for 1hr. (a) Describes the process for sample preparation. (b,c) show the as-measured Sn amounts (ppm) determined by ICP-OES. (d,e) Show the same data as atomic percentage relative to the measured Au content .

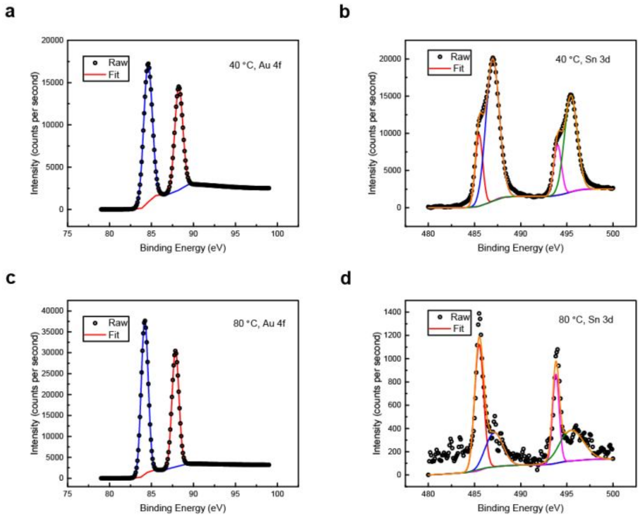

Figure S29. X-ray photoelectron spectra (a) 40% Sn added Au 4f, (b) 40% Sn added Sn 3d nanoparticles synthesized at 40 °C. (c) 40% Sn added Au 4f, and 40% Sn added Sn 3d nanoparticles synthesized at 80 °C.

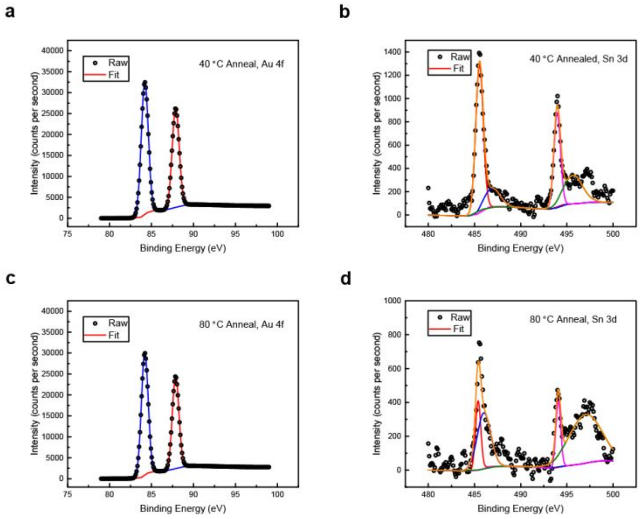

Figure S30. X-ray photoelectron spectra (a) 40% Sn added Au 4f, (b) 40% Sn added Sn 3d nanoparticles synthesized at 40 °C and annealed at 80 °C for 1 hour. (c) 40% Sn added Au 4f, and 40% Sn added Sn 3d nanoparticles synthesized at 80 °C and annealed at 80 °C for 1 hour.

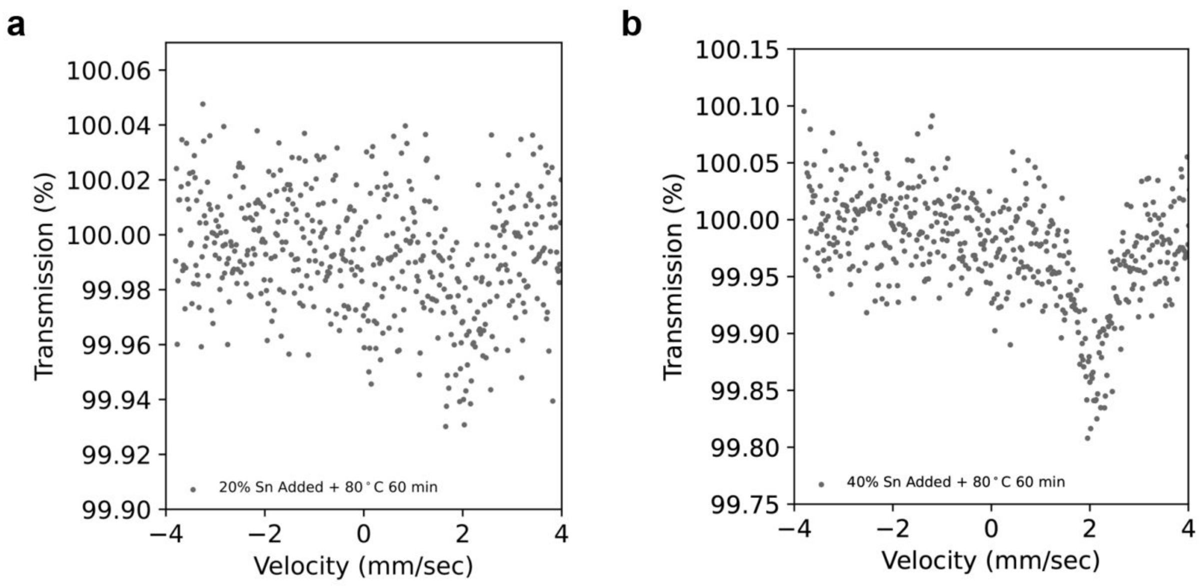

Figure S31. Unbinned Mössbauer spectra following background subtraction and velocity calibration for (a) 20% Sn-added and (b) 40% Sn-added Au-Sn nanoparticles as-synthesized at 60 °C and annealed at 80 °C for one hour scanned between +/- 4 mm/s. These spectra correspond to those shown in Figure 4b,d.

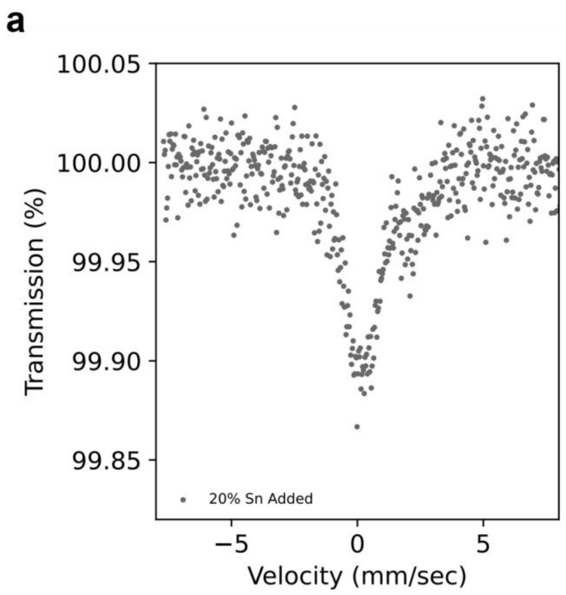

Figure S32. Unbinned Mössbauer spectra following background subtraction and velocity calibration for (a) 20% Sn-added and (b) 40% Sn-added Au-Sn nanoparticles as-synthesized at 60 °C scanned between +/- 8 mm/s. These spectra correspond to those shown in Figure 4a,c.

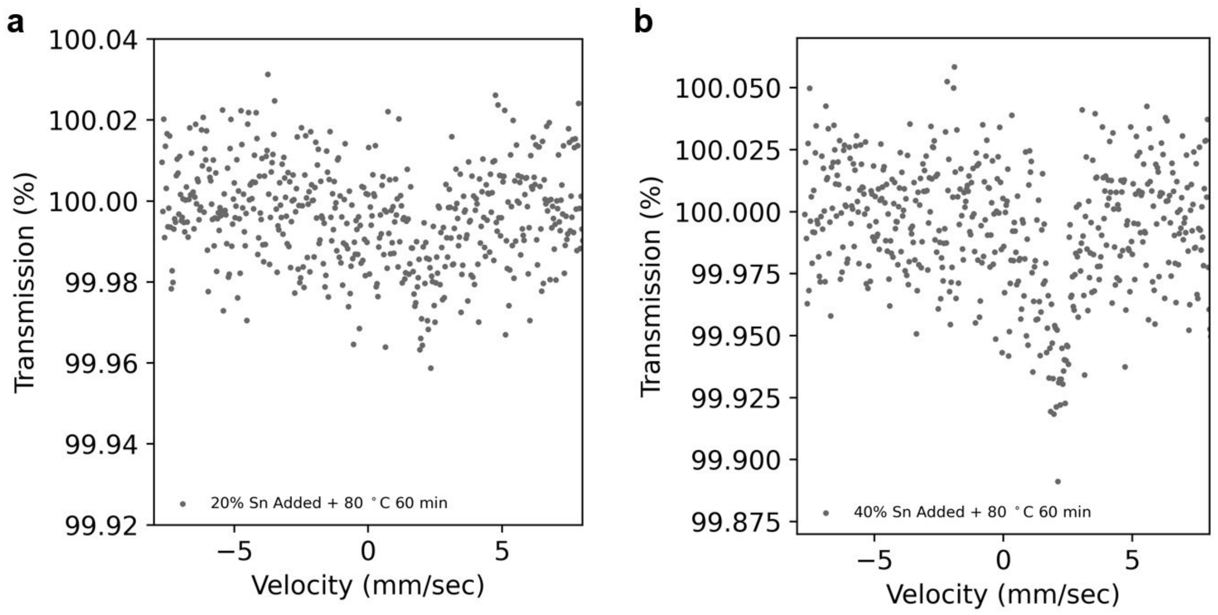

Figure S33. Unbinned Mössbauer spectra following background subtraction and velocity

calibration for (a) 20% Sn-added and (b) 40% Sn-added Au-Sn nanoparticles synthesized at 60 °C and annealed at 80 °C for one hour scanned between +/- 8 mm/s.

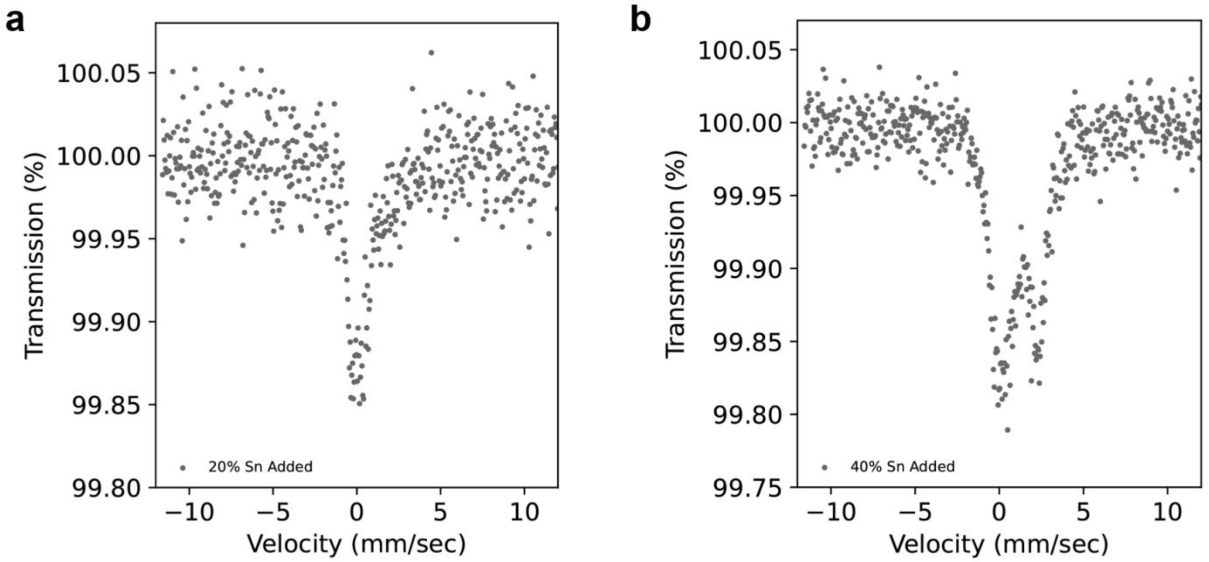

Figure S34. Unbinned Mössbauer spectra following background subtraction and velocity calibration for (a) 20% Sn-added and (b) 40% Sn-added Au-Sn nanoparticles as-synthesized at 60 °C scanned between +/- 12 mm/s. These spectra were measured for samples prepared separately from those whose spectra are shown in Figure 4, S30.

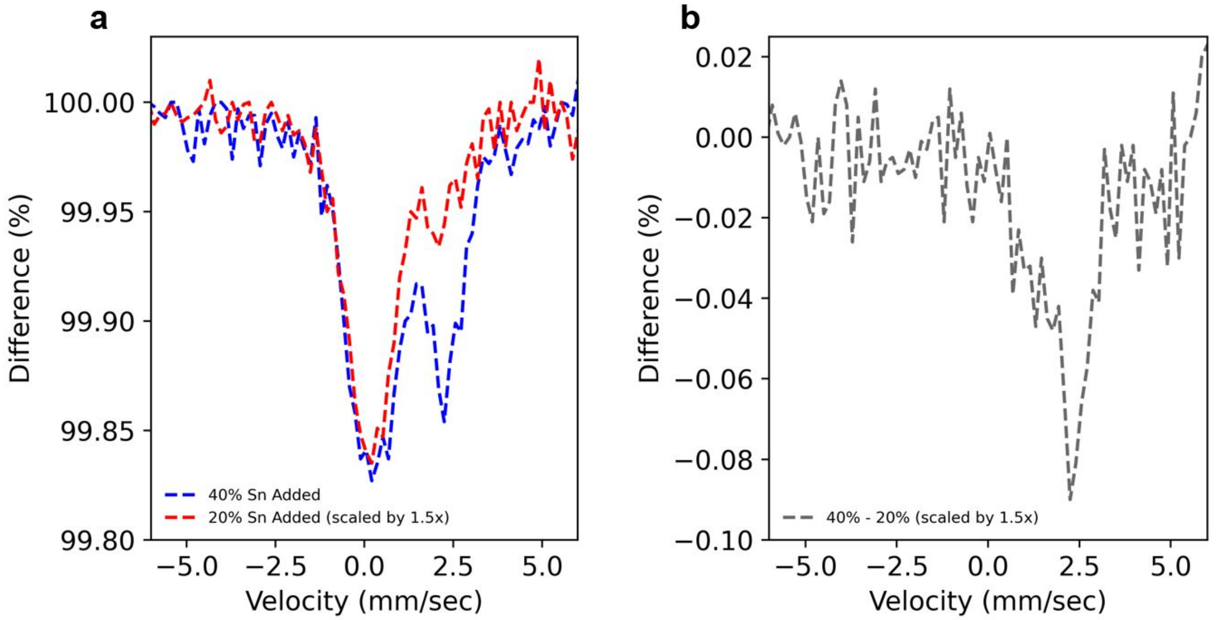

Figure S35. a) Binned Mössbauer spectra following background subtraction and velocity calibration for 40% Sn-added and 20% Sn-added (scaled by 1.5x such that the minima of both peaks are equal) Au-Sn nanoparticles as-synthesized at 60 °C. b) Plot of the difference between the 40% and scaled 20% Sn spectra in (a). It can be seen that the main peak for 40% is broader in the positive direction compared to the 20% scaled spectrum and the difference reveals a peak at 0.6 mm/s, implying there is no or minimal contribution of a third Lorentzian in the 20% as-synthesized sample.

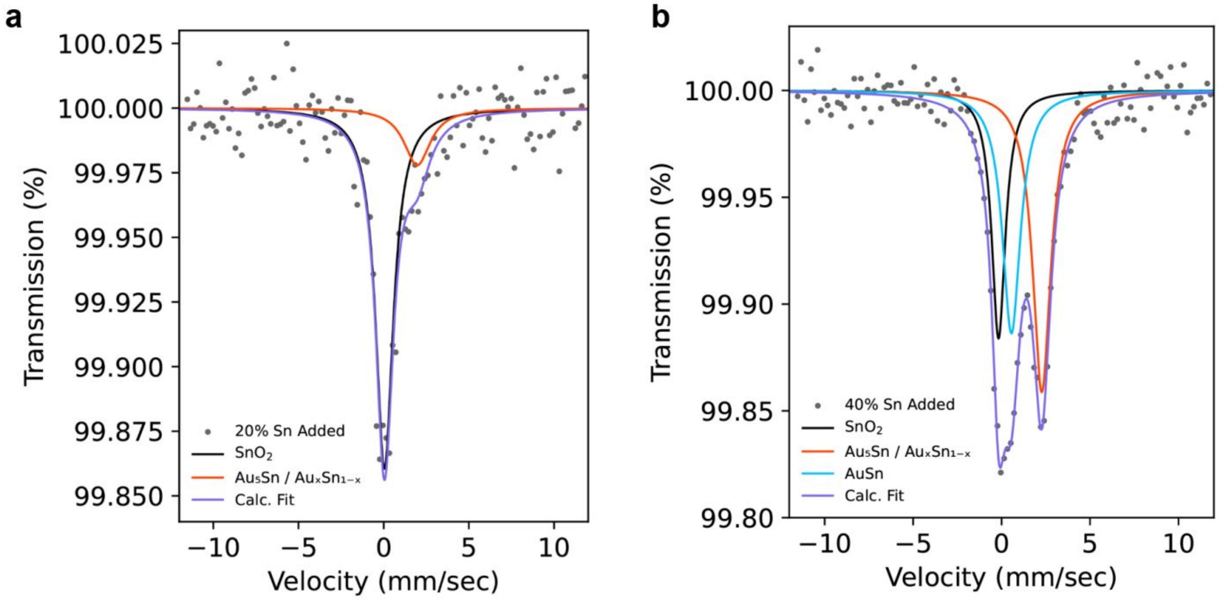

Figure S36. Binned Mössbauer spectra for (a) 20% and (b) 40% Sn-added Au-Sn nanoparticles as-synthesized at 60 °C scanned between +/- 12 mm/s. These spectra correspond to those shown in Figure S32.

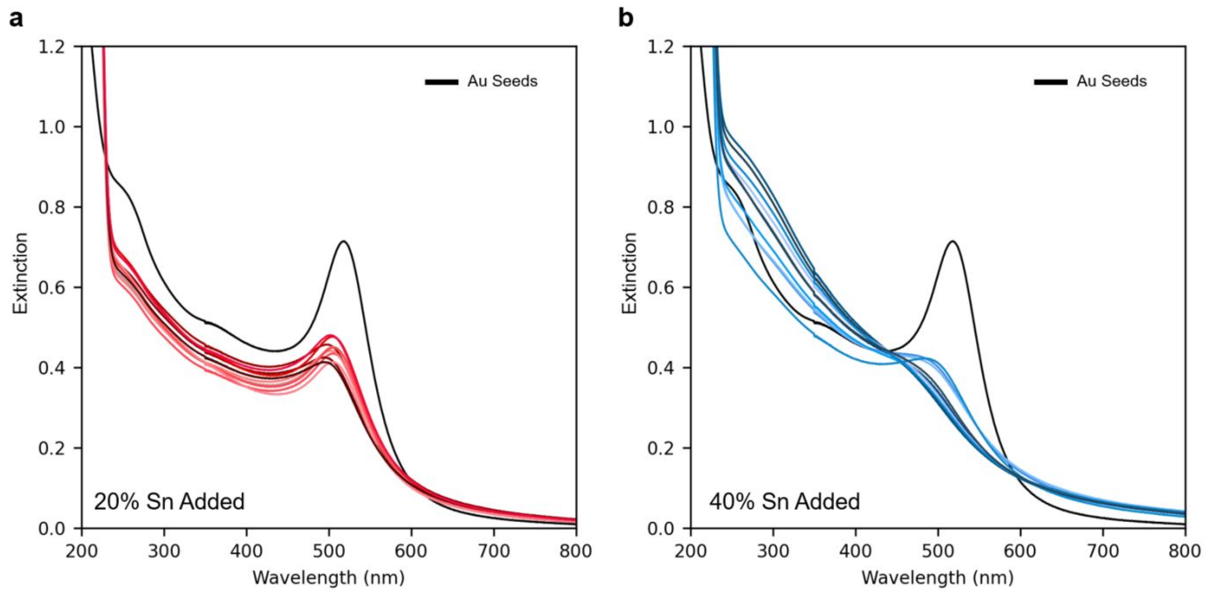

Figure S37. Unnormalized extinction spectra for n=10 as-synthesized (a) 20% and (b) 40% Au-Sn nanoparticles synthesized at 60 °C.

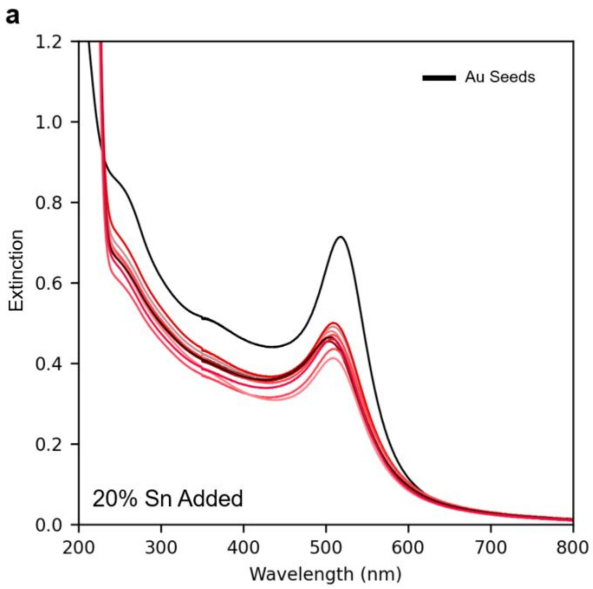

Figure S38. Unnormalized extinction spectra for n=10 annealed (a) 20% and (b) 40% added Au-Sn nanoparticles synthesized at 60 °C and annealed at 80 °C for one hour.

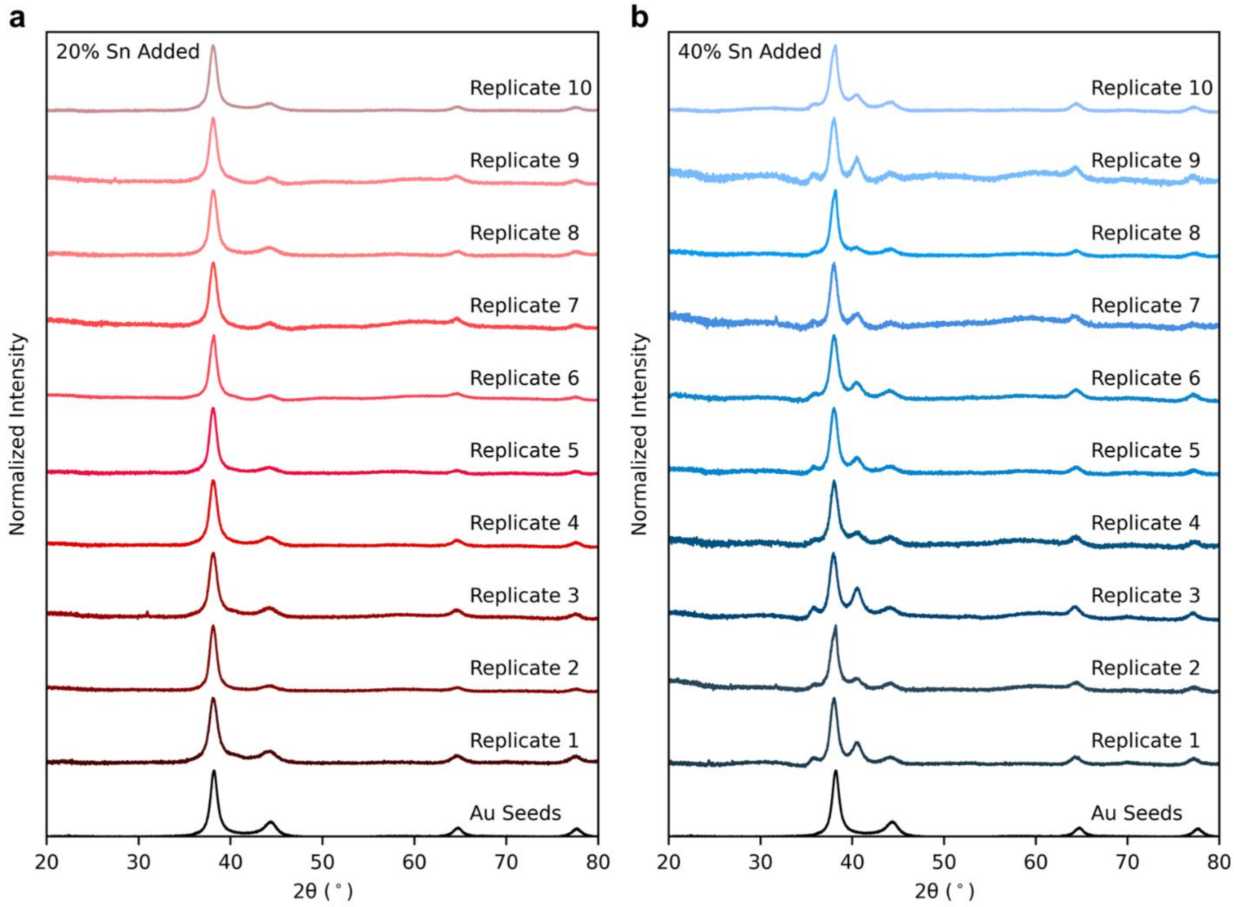

Figure S39. Replicate n=10 XRD experiments on (a) 20% and (b) 40% added Au-Sn synthesized at 60 °C after annealing at 80 °C for 1 hour.

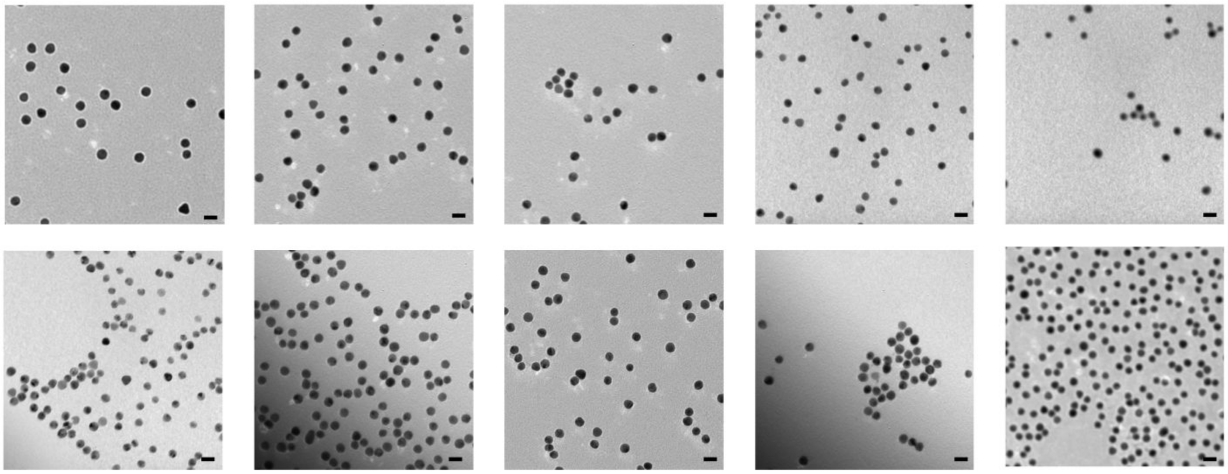

Figure S40. Representative transmission electron microscopy of 10 replicate experiment 20% Sn-added Au-Sn nanoparticles synthesized at 60 °C after annealing at 80 °C for 1 hour. Scale bars are 20 nm.

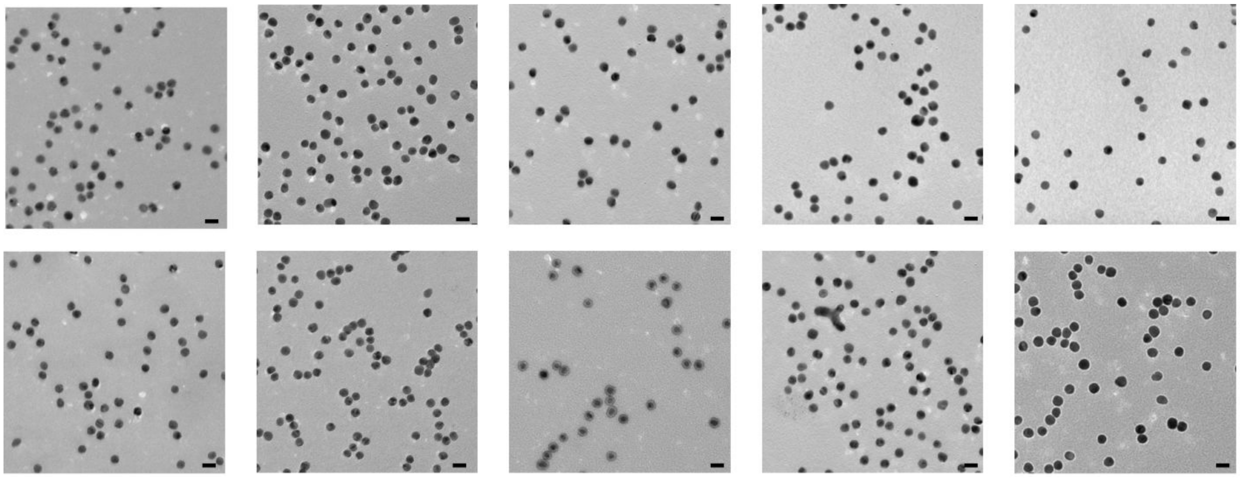

Figure S41. Representative transmission electron microscopy of 10 replicate experiment 40% Sn-added Au-Sn nanoparticles synthesized at 60 °C after annealing at 80 °C for 1 hour. Scale bars are 20 nm.

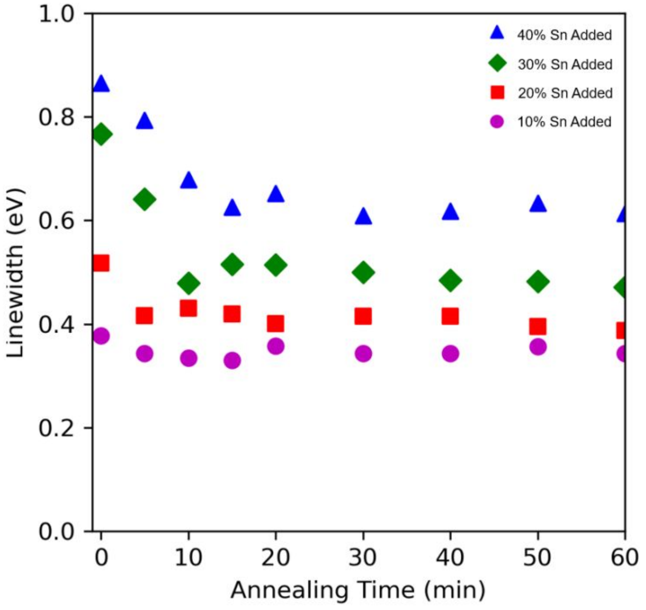

Figure S42. Linewidth broadening vs. LSPR peak wavelength for 10%, 20%, 30%, and 40% Sn-added nanoparticles synthesized at 60°C and annealed for 0, 2.5, 5.0, 7.5, 10, 15, 20, 30, and 60 minutes at 80 °C.

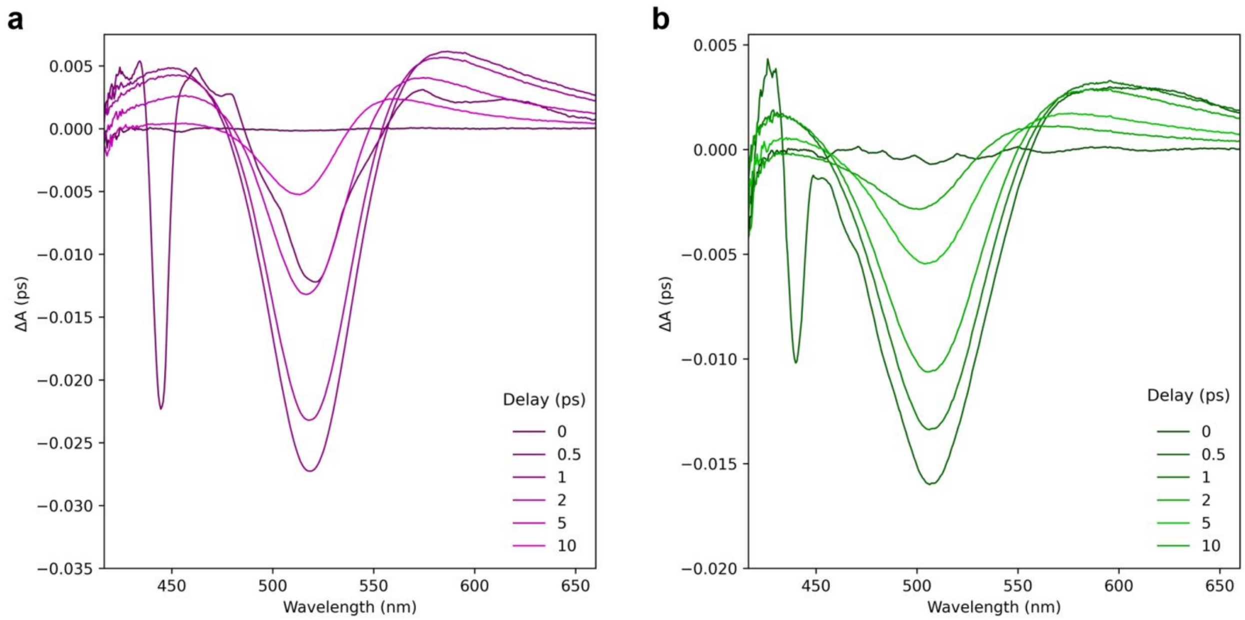

Figure S43. High power transient absorption bleach spectra for (a) 10% and (b) 30% Sn-added annealed nanoparticles synthesized at 60 °C after annealing at 80 °C for 1 hour.

References

1. Turkevich, J., Stevenson, P.C., Hillier, J. The Formation of Colloidal Gold *J. Phys. Chem.* 1953 **57**, 670-673.
2. Branco, A.J., Dawes, S.S., Mason, N. L., Fonseca Guzman, M.V., King, M.E., Ross, M.B. Synthesis of gold-tin alloy nanoparticles with tunable plasmonic properties *STAR Protocols* 2023 **4**, 102410.
3. Fonseca Guzman, M. V., King, M. E., Mason, N. L., Sullivan, C. S., Jeong, S., Ross, M. B., Plasmon manipulation by post-transition metal alloying. *Matter* **2023,** *6* (3), 838-854.
4. Fonseca Guzman, M. V., Ross, M. B., Radiative Contributions Dominate Plasmon Broadening for Post-Transition Metals in the Ultraviolet. *J. Phys. Chem. C* **2021,** *125* (35), 19428-19437.
5. Blaber, M. G., Henry, A.-I., Bingham, J. M., Schatz, G. C., Van Duyne, R. P., Lspr Imaging of Silver Triangular Nanoprisms: Correlating Scattering with Structure Using Electrodynamics for Plasmon Lifetime Analysis. *J. Phys. Chem. C* **2011**, *116*, 393-403.
6. Hartland, G. V., Optical Studies of Dynamics in Noble Metal Nanostructures. *Chem. Rev.* **2011**, *111*, 3858-3887.
7. Bosbach, J., Hendrich, C., Stietz, F., Vartanyan, T., Träger, F., Ultrafast Dephasing of Surface Plasmon Excitation in Silver Nanoparticles: Influence of Particle Size, Shape, and Chemical Surrounding. *Phys. Rev. Lett.* **2002**, *89*.
8. Kreibig, U., Small Silver Particles in Photosensitive Glass: Their Nucleation and Growth. *Appl. Phys.* **1976**, *10*, 255-264.
9. Zorić, I., Zäch, M., Kasemo, B., Langhammer, C., Gold, Platinum, and Aluminum Nanodisk Plasmons: Material Independence, Subradiance, and Damping Mechanisms.

ACS Nano **2011**, *5*, 2535-2546.

10. Foerster, B., Spata, V. A., Carter, E. A., Sönnichsen, C., Link, S., Plasmon Damping Depends on the Chemical Nature of the Nanoparticle Interface. *Sci. Adv.* **2019**, *5*.
